# Chemical and structural characterization of a model Post-Termination Complex (PoTC) for the ribosome recycling reaction: Evidence for the release of the mRNA by RRF and EF-G

**DOI:** 10.1371/journal.pone.0177972

**Published:** 2017-05-24

**Authors:** Nobuhiro Iwakura, Takeshi Yokoyama, Fabio Quaglia, Kaoru Mitsuoka, Kazuhiro Mio, Hideki Shigematsu, Mikako Shirouzu, Akira Kaji, Hideko Kaji

**Affiliations:** 1 Department of Biochemistry and Molecular Biology, Thomas Jefferson University, Jefferson Medical College, Philadelphia, Pennsylvania, United States of America; 2 Department of Microbiology, Perelman School of Medicine, University of Pennsylvania, Philadelphia, Pennsylvania, United States of America; 3 Division of Structural and Synthetic Biology, RIKEN Center for Life Science Technologies, Yokohama, Japan; 4 University of Camerino, School of Biosciences and Veterinary Medicine, Camerino, Italy; 5 Research Center for Ultra-High Voltage Electron Microscopy, Osaka University, Osaka, Japan; 6 Molecular Profiling Research Center for Drug Discovery and OPERANDO Open Innovation Laboratory, National Institute of Advanced Industrial Science and Technology, Tokyo, Japan; John Curtin School of Medical Research, AUSTRALIA

## Abstract

A model Post-Termination Complex (PoTC) used for the discovery of Ribosome Recycling Factor (RRF) was purified and characterized by cryo-electron microscopic analysis and biochemical methods. We established that the model PoTC has mostly one tRNA, at the P/E or P/P position, together with one mRNA. The structural studies were supported by the biochemical measurement of bound tRNA and mRNA. Using this substrate, we establish that the release of tRNA, release of mRNA and splitting of ribosomal subunits occur during the recycling reaction. Order of these events is tRNA release first followed by mRNA release and splitting almost simultaneously. Moreover, we demonstrate that IF3 is not involved in any of the recycling reactions but simply prevents the re-association of split ribosomal subunits. Our finding demonstrates that the important function of RRF includes the release of mRNA, which is often missed by the use of a short ORF with the Shine-Dalgarno sequence near the termination site.

## Introduction

Until recently, protein synthesis was thought to consist of three steps, initiation, elongation, and termination. The concept of an additional step of protein synthesis was initiated by the discovery of a protein factor dedicated to the release of ribosomes from the mRNA of the post-termination complex (PoTC) [1]. For a review of early history, see [2]. This factor was named “ribosome releasing factor (RRF)” because RRF, in cooperation with EF-G (elongation factor G) releases mRNA from the PoTC [1]. Later, when we found that RRF is an essential factor [3], we realized that the reason RRF releases mRNA from the ribosome of the PoTC is to re-use the ribosomes and mRNA for the new round of translation and thus renamed it “ribosome recycling factor”[3]. The first recognition of the importance of this factor by another laboratory was the finding by the Buckingham and Ehrenberg group that the suppresser of temperature sensitive peptidyl tRNA hydrolase (an essential factor) lies on the promoter region of *frr* (gene coding for RRF [4]). It appeared that a reduced amount of RRF would prevent the lethal effect of missing peptidyl tRNA hydrolase. The general interest in this factor was heightened by the finding that RRF is a nearly perfect structural mimic of tRNA [5]. The reason for this similarity is that both RRF and tRNA move in the inter ribosomal subunits space to function [6, 7]. During the initial characterization of RRF, it was clear that it functions to release tRNA as well as mRNA [8]. The third function, the splitting of 70S ribosomes into their subunits, was suggested much later by the use of a short ORF containing three codons situated near a Shine Dalgarno (SD) sequence for the ribosome binding [9]. The splitting of 70S ribosomes by RRF and EF-G was established through further *in vitro* experiments by three laboratories [10–12].

Despite the general consensus of the importance of RRF and its ribosome recycling role, as described above, there have been considerable differences regarding the exact nature of its substrate, the reaction product, and the order of events (tRNA release, mRNA release and splitting of the 70S ribosome) [8–11, 13–16].

The original substrate of the RRF assay had bound tRNAs that were partially released by puromycin and EF-G alone [8]. In this paper, we prepared the new substrate, which was free from tRNA releasable by EF-G and/or puromycin. We then rigorously established the structure and chemical characteristics of this PoTC. We conclude that the new substrate prepared in this study has mostly one tRNA, at the P/E or P/P position. From this point on, when we refer to the PoTC, we refer to this substrate with one tRNA. The PoTC we used in past publications with more than one tRNA will be referred to as the “crude-PoTC”. Using this well-defined model substrate, we determined the reaction product to consist of mRNA, tRNA, and ribosomal subunits. The release of tRNA occurs first, followed by mRNA release and splitting of 70S ribosomes with almost the same rate.

## Results

### Preparation of the PoTC from crude PoTC

In our previous studies, as a substrate for the ribosome recycling reaction, we used polysomes isolated directly from the cells in the mid-log phase at various stages of protein synthesis (See S1A Fig). After isolation of the polysomes from the cells, we moved peptidyl tRNA from the A-site to the P-site by EF-G (S1A to S1B Fig), followed by the removal of the nascent peptidyl group from the translocated peptidyl tRNA by the addition of puromycin (S1B to S1C Fig). In this assay system, the amount of tRNA released does not represent the true amount of tRNA released during the disassembly of the PoTC by RRF and EF-G [8]. In fact, a portion of the tRNA released in this assay represents the tRNA released during the puromycin and translocation step prior to ribosome recycling.

**Fig 1:**
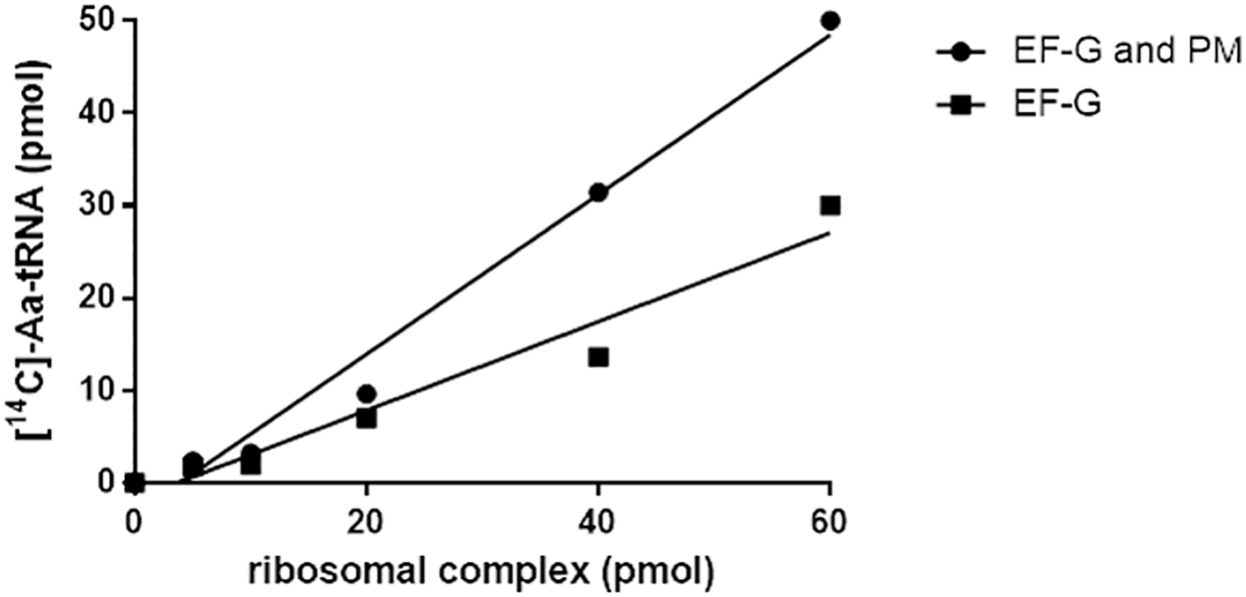
Release of tRNA during the preparation of the PoTC as schematically described in S1 Fig. Various amounts of polysomes were incubated with 2 μM EF-G and 1 mM GTP and isolated (A in S1 Fig). Total reaction mixture was 50 μl. The polysomes (B in S1 Fig) thus isolated were incubated with 50 μM puromycin in buffer R. The tRNA released during these operations was isolated by passing through nitrocellulose membrane (0.45 nm). Transfer RNA thus obtained was measured by aminoacylation with a mixture of [^14^C] amino acids. The Y-axis shows the released tRNA (pmols) calculated from [^14^C]-aminoacyl-tRNA (cpm) as described in Materials and methods and the supplement. Squares, tRNA released by EF-G/GTP; circles, tRNA released by puromycin and EF-G/GTP.

To overcome this issue, we pre-incubated the polysomes extracted from the growing cells with EF-G and puromycin and re-isolated them using a sucrose gradient. To prepare this model Post-Termination Complex (PoTC), we treated the polysomes first with EF-G. In the experiment shown in Fig 1, various amounts of polysomes were treated with EF-G, and the reaction mixture was filtered through a Millipore filter to trap only the ribosomes, allowing us to separate released tRNAs from the polysomes. The tRNA thus released was measured by charging with a ^14^C amino acid mixture. It is clear from this figure that tRNA is released by EF-G and puromycin alone without RRF. This result indicates that the original substrate used contains extra tRNA that can be released during the routine RRF assay. These results are consistent with the originally reported findings [8] and indicate that the substrate that we have been using contains an extra tRNA that does not participate in the RRF reaction.

### Cryo-EM characterization of the PoTC

**Fig 2:**
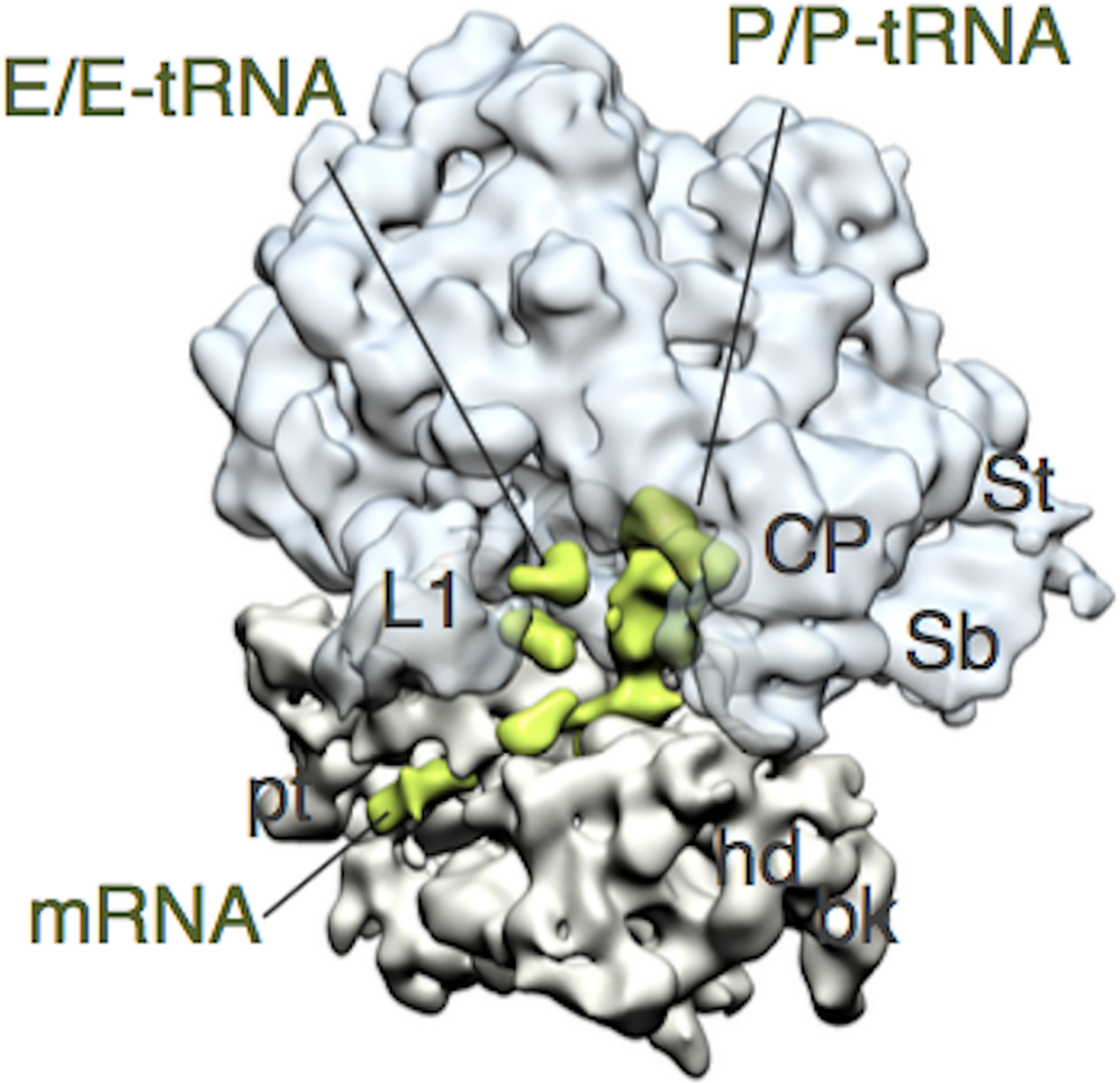
Cryo-EM structure of the ribosome from polysomes (EMD-9533). Cryo-EM structure reconstructed from the images of purified *E*. *coli* endogenous polysomes. Sixty-three percent of the total particles was used for the reconstruction. Reconstructed ribosome is in the unrotated state containing densities in the P site, E site and mRNA path. Color code: 50S, aqua; 30S, light brown; tRNAs and mRNA, green. Landmarks of ribosome: CP, central protuberance; L1, L1 stalk; St, L7/12 stalk; Sb, L7/12 stalk base; hd, head; bk, beak; pt, platform.

Although it has been over 40 years since RRF was discovered [17], the nature of the substrate of this enzyme has remained controversial. The major point of discrepancy in the literature is the number and position of the ribosome-bound tRNAs. Using crude PoTC derived from growing cells, we proposed that there are two tRNAs at the P/P and E/E sites [14] based on the report that each ribosome of the polysomes contains approximately two tRNAs per ribosome [11, 13, 18]. This result was confirmed by cryo-EM studies on polysomes, as shown in Fig 2. On the other hand, some publications appear to support the PoTC containing one tRNA at the P-site [9, 10, 19, 20].

**Fig 3:**
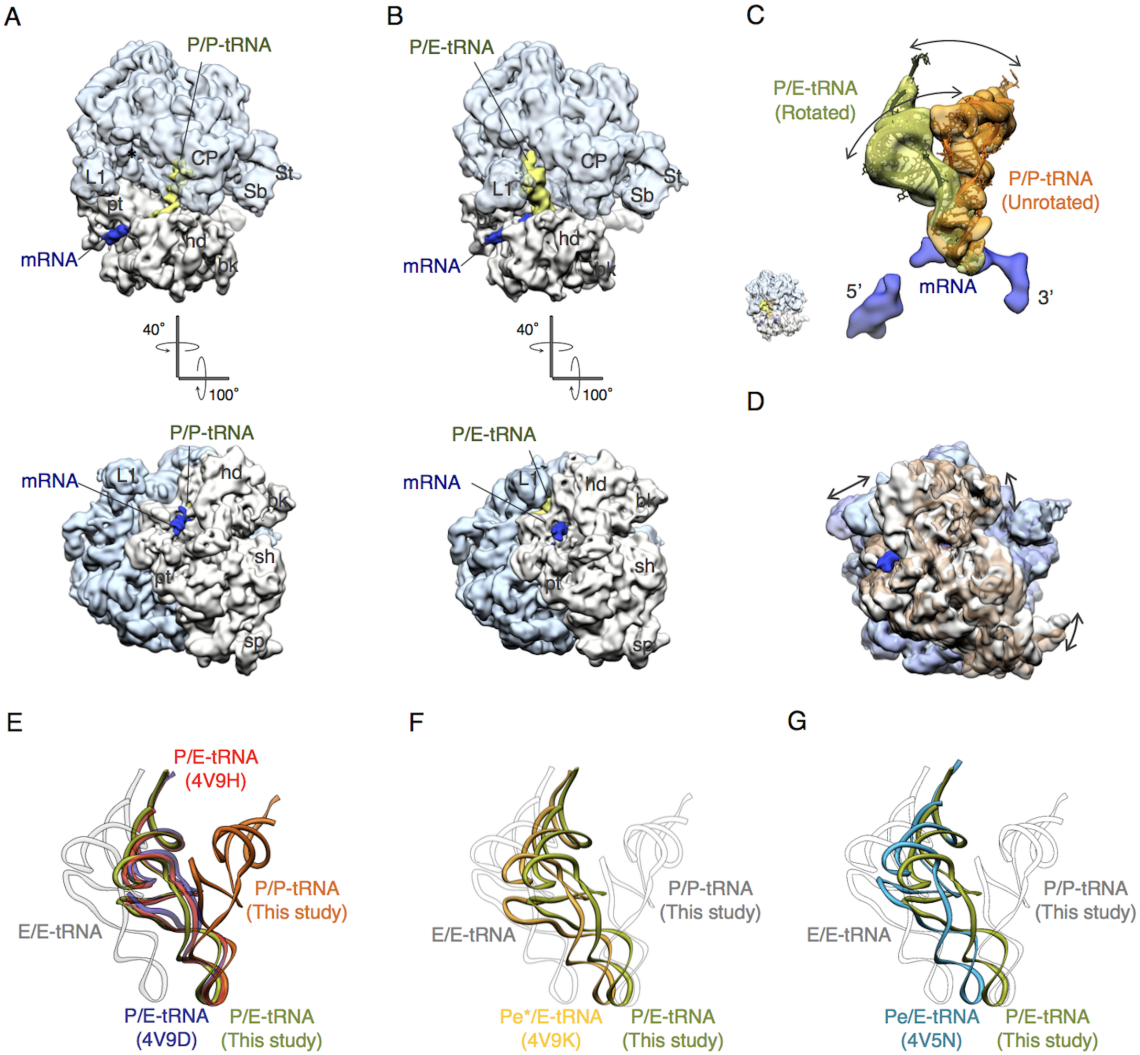
Cryo-EM structures of the PoTC. (A and B) cryo-EM structures reconstructed from images of model PoTC, prepared by treating the purified *E*. *coli* endogenous polysomes with Puromycin. **(A**) The PoTC in unrotated state (**EMD-9531**) containing one tRNA molecule in P site (green) and mRNA (blue) in the mRNA path. (B) The PoTC in the rotated state (**EMD-9530**) containing one tRNA molecule in the P/E hybrid state (green) and mRNA (blue) in the mRNA path in the 30S subunit. Segmented densities of all components of the PoTC in this figure are shown at the same contour level. Color code: 50S, aqua; 30S, light brown; tRNA, green; mRNA, blue. Landmarks of ribosome: CP, central protuberance; L1, L1 stalk; St, L7/12 stalk; Sb, L7/12 stalk base; hd, head; bk, beak; pt, platform; sh, shoulder; sp, spur. (C) Fluctuation of tRNA in two different positions in PoTC in conjunction with its configurational rearrangement. Comparison of tRNA positions between two cryo-EM structures obtained from the PoTC specimen. The 30S subunits were used as guides for the precise alignment of the coordinates of the two structures. The anticodon stem loops of the tRNA densities share the same position on 30S subunits, which is the P site. By contrast, the elbow regions and CCA ends of the tRNAs are widely rotated and shifted between the two structures. Cryo-EM structure of mRNA segmented from the rotated PoTC is also shown here. (D) Rotational motion of 30S is shown by the alignment of the 50S portions of two structures from the PoTC specimen. Color code: 50S in rotated state, aqua; 30S in rotated state, light brown; 50S in unrotated state, semitransparent purple; 30S in unrotated state, semitransparent brown. (E) Comparison of tRNA positions in the PoTC with published intermediate positions. Two tRNA positions in the PoTC occur in this study: the P/E hybrid state (green) and classical P-tRNA (orange) are compared with previously reported P/E-tRNAs (PDB ID: 4V9H [20], red; PDB ID: 4V9D [21], purple). (F) P/E-tRNA (green) in the PoTC is compared with tRNA in the Pe*/E intermediate position (PDB ID: 4V9K [22], yellow). (G) P/E-tRNA (green) in the PoTC is compared with tRNA in the Pe/E intermediate position (PDB ID: 4V5N [23], blue).

To settle the confusion, we decided to perform cryo-EM single particle analysis on the PoTC. The cryo-EM grids were prepared, and the samples were examined at 300 kV accelerating voltage at liquid helium temperature (4K). Through the image analysis and reconstructions, two cryo-EM structures of a single PoTC in two different conformational states were obtained (Fig 3A and 3B and S2 Fig). In the first conformational state (Fig 3A), the ribosome forming the PoTC was in the unrotated state. This cryo-EM structure contains distinct densities attributed to mRNA (blue) and P/P-tRNA (green). It should be noted that L1 stalk of this structure is open toward the solvent side implying that E site of this ribosome is empty. In the second conformational states (Fig 3B), the ribosome is in the rotated state and contains mRNA (blue) and one tRNA molecule (green) in the P/E hybrid state. L1 stalk of this structure is closed and interacted with the elbow region of P/E-tRNA. From the cryo-EM results obtained here, we conclude that each PoTC molecule contains one tRNA. Depending on the global configuration of the ribosome, tRNA is positioned in the P/P or P/E hybrid state (compare Fig 3A and 3B). In addition, it was visually confirmed that the PoTC is attached to mRNA, since mRNA density was clearly observed in the mRNA path on the 30S subunit in the single PoTC (Fig 3C).

### Fluctuation of tRNA between P/P and P/E on the PoTC and comparison with tRNA during chain elongation

Transfer RNA densities on the PoTC structures at P/E and at P/P were compared by alignment with the 30S portions of the structures, which were used as guides. The anti-codon stem loop region of both tRNA structures share the same site (P site) on the 30S subunit (Fig 3C). In addition, the overall configuration of the PoTC containing P/E-tRNA is rotated (Fig 3D, aqua, 50S subunit and light brown, 30S subunit), while the PoTC containing P/P-tRNA are unrotated (Fig 3D, semi-transparent purple, 50S subunit and semitransparent brown, 30S subunit), indicating that the tRNA motion is coupled with the global conformational change of the PoTC. The P/P and P/E positions of the tRNAs reported in this study correspond to the previous observations in another study (see Fig 3E) [20–23]. In addition, comparison with the structure of our post-termination complex with the translocation intermediate reported by [20] confirmed that the tRNA position of our PoTC is indeed at the P/E site. However, it is noteworthy that the cryo-EM structure shows more of the P/E tRNA, while the crystal structure shows mostly P/P tRNA.

Recently, several intermediate positions of tRNA between the P/E hybrid state and classical E-tRNA were reported. Two intermediate tRNA positions are compared with our P/E-tRNA: one is the Pe*/E position from PDB ID: 4V9K [22] (Fig 3F), and the other is the Pe/E position from [23] PDB ID: 4V5N (Fig 3G). As a result of these comparisons, we conclude that the tRNA in our PoTC is situated in a different position from any of the intermediates such as Pe/E or Pe*/E.

### Characterization of the PoTC prepared above through biochemical means—Number of tRNAs per PoTC

**Fig 4:**
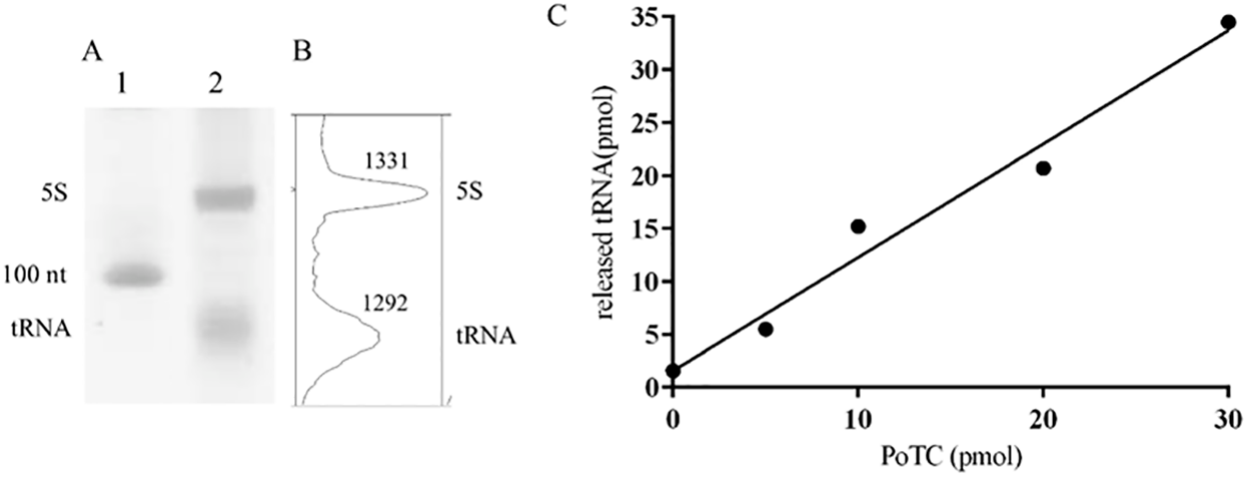
Additional evidence that every PoTC contains one tRNA: Comparison with 5S RNA. The amounts of tRNA and 5S rRNA in the PoTC were compared by UREA-PAGE. (A) Lane 1, size marker; Lane 2, total RNA from 5 pmol of PoTC. (B) The density analysis from lane 2 in (A). The top and bottom peaks correspond to 5S rRNA and tRNA, respectively. The numbers on each peak are generated by ImageJ analysis of the density of 5S rRNA and tRNA. (C) The released tRNA at low Mg^2+^ concentrations from various amounts of PoTC in buffer L was isolated and aminoacylated as described under “tRNA measurement by aminoacylation” in the Materials and methods section. After conversion from cpm to pmol, the amount of tRNA (Y-axis) was plotted against the amount of PoTC (X-axis).

The preceding results settle the nature of the PoTC in terms of structural information. It would be desirable to characterize the PoTC biochemically. In the experiments shown in Fig 4A and 4B, the PoTC prepared as above was subjected to RNA extraction. Because one molecule of 5S RNA exists per ribosome, it was reasoned that if there is one tRNA per PoTC, as suggested by the cryo-EM studies, one would expect equal moles of 5S RNA and tRNA. Due to the similarity of molecular weights, the behavior of these RNAs in denaturing gel electrophoresis should be similar. Accordingly, we have analyzed the RNA extracted from the PoTC. As shown in Fig 4A, two small RNAs are clearly present in the PoTC. Fig 4B shows the densitometry of these two bands, indicating that they are present in equal amounts. Moreover, the PoTC was subjected to low (1 mM) Mg^2+^ to release all the bound tRNA. The released tRNA was subjected to aminoacylation with ^35^S methionine. We estimated the amount of picomoles of tRNA per picomoles of PoTC (S5A and S5B Fig). It is clear from Fig 4C that approximately one molecule of tRNA is bound to the PoTC.

To confirm that treatment of PoTC with 1 mM Mg^2+^ indeed releases all tRNA bound to the PoTC, total RNA was extracted from the treated PoTC and subjected to gel electrophoresis. As shown in S3C Fig, there was no residual tRNA on the ribosome after the low Mg^2+^ treatment. We conclude that the PoTC contains one tRNA.

### Characterization of products of the RRF/EF-G reaction in the PoTC—Cryo EM studies

**Fig 5:**
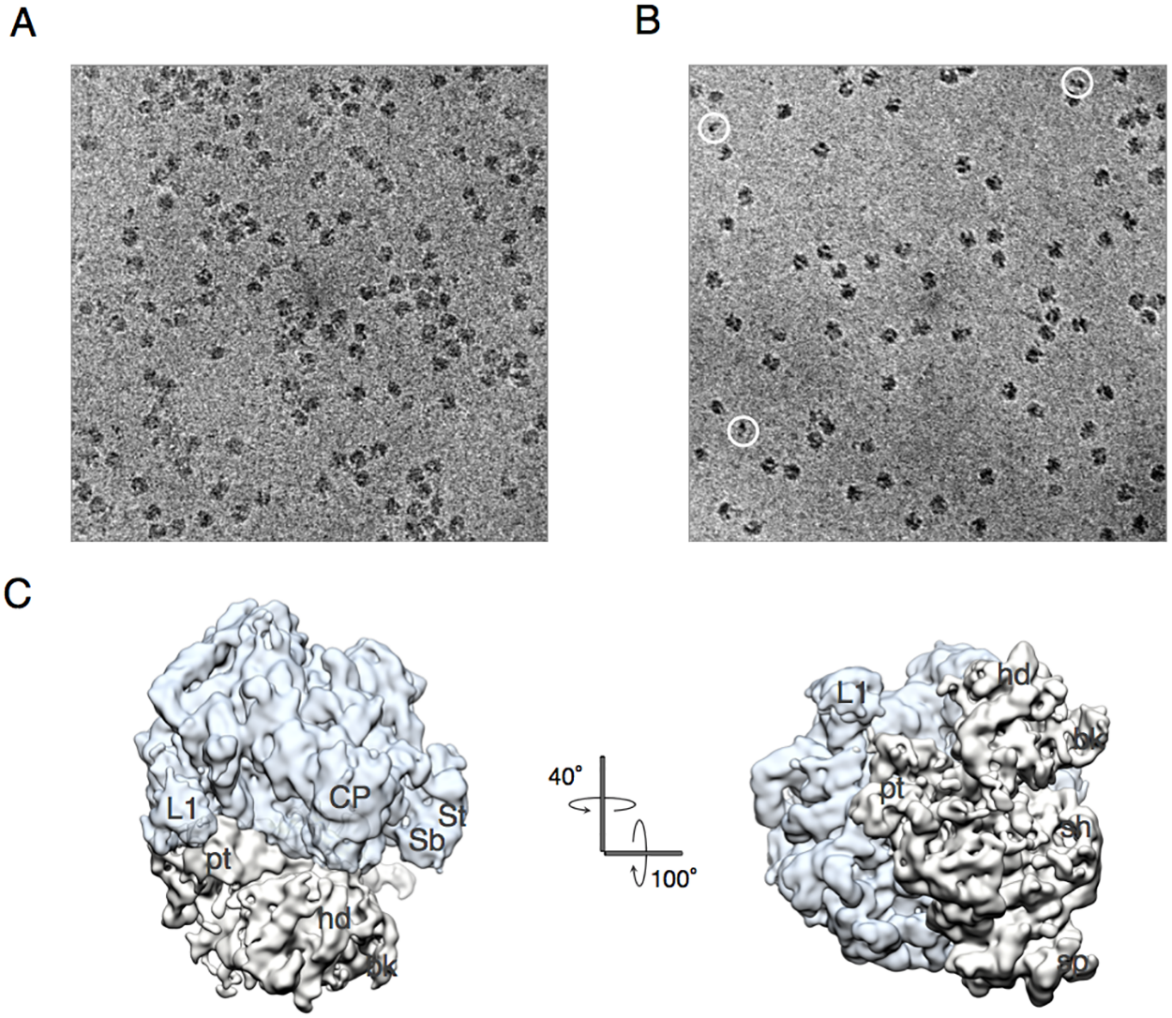
mRNA release from the PoTC and splitting of ribosomal subunits by RRF and EF-G. (A) PoTC (100 nM) before the reaction and (B) the reaction product in frozen-hydrated states was observed with a transmission electron microscope (JEM1230, JEOL) operated at 120 kV at liquid nitrogen temperature. (B) Reaction products consist mainly reassociated 70S. It is possible to see dissociated 30S on the cryo-EM grid (see particles in white circles). Compared with the PoTC (A), 70S ribosome particles evenly spread over the thin carbon layer due to the release of ribosomes from mRNA by the ribosome recycling reaction. Specimens were observed at 40,000-fold magnification in a -3.5 μm defocused condition. Images were obtained with 1 K X 1 K fast scan CCD (charge coupled device) camera (TVIPS: Tietz Video and Image Processing Systems). (C) Single-particle analysis of cryo-EM images of the PoTC after the RRF reaction. Figure shows vacant 70S ribosomes formed after the recycling reaction (**EMD-9532**). The 70S ribosome does not have any densities ascribed to released components (tRNA and mRNA), which are clearly observed in the PoTC (Fig 3A and 3B). Color code: 50S, aqua; 30S, light grey. Landmarks of ribosome: CP, central protuberance; L1, L1 stalk; St, L7/12 stalk; Sb, L7/12 stalk base; hd, head; bk, beak; pt, platform; sh, shoulder; sp, spur.

To observe the organization of the PoTC and to investigate how it changes upon reaction with RRF and EF-G, ribosome specimens were examined in a frozen-hydrated condition using a transmission electron microscope operated at a lower accelerating voltage (120 kV). This approach permitted us to visualize specimens at higher contrast (Fig 5A and 5B). Before the RRF/EF-G reaction, the ribosomes of the PoTC formed a cluster by attaching to the mRNA (Fig 5A). The organization of ribosomes was similar to the previously reported translating *E*. *coli* polysomes [24]. However, after the recycling reaction, the observed PoTC clusters were dissolved, causing the diffusion of 70S ribosomes over the grid (Fig 5B). RRF releases mRNA from ribosomes, resulting in the conversion of the polysome into monosomes. It appears that there are more ribosomes in 5A than in 5B because polysomes are much longer than the monosomes and more easily adsorbed on the thin carbon layer of the grid.

### Structural evidence for RRF being the recycling factor that releases mRNA and tRNA from the PoTC

**Fig 6:**
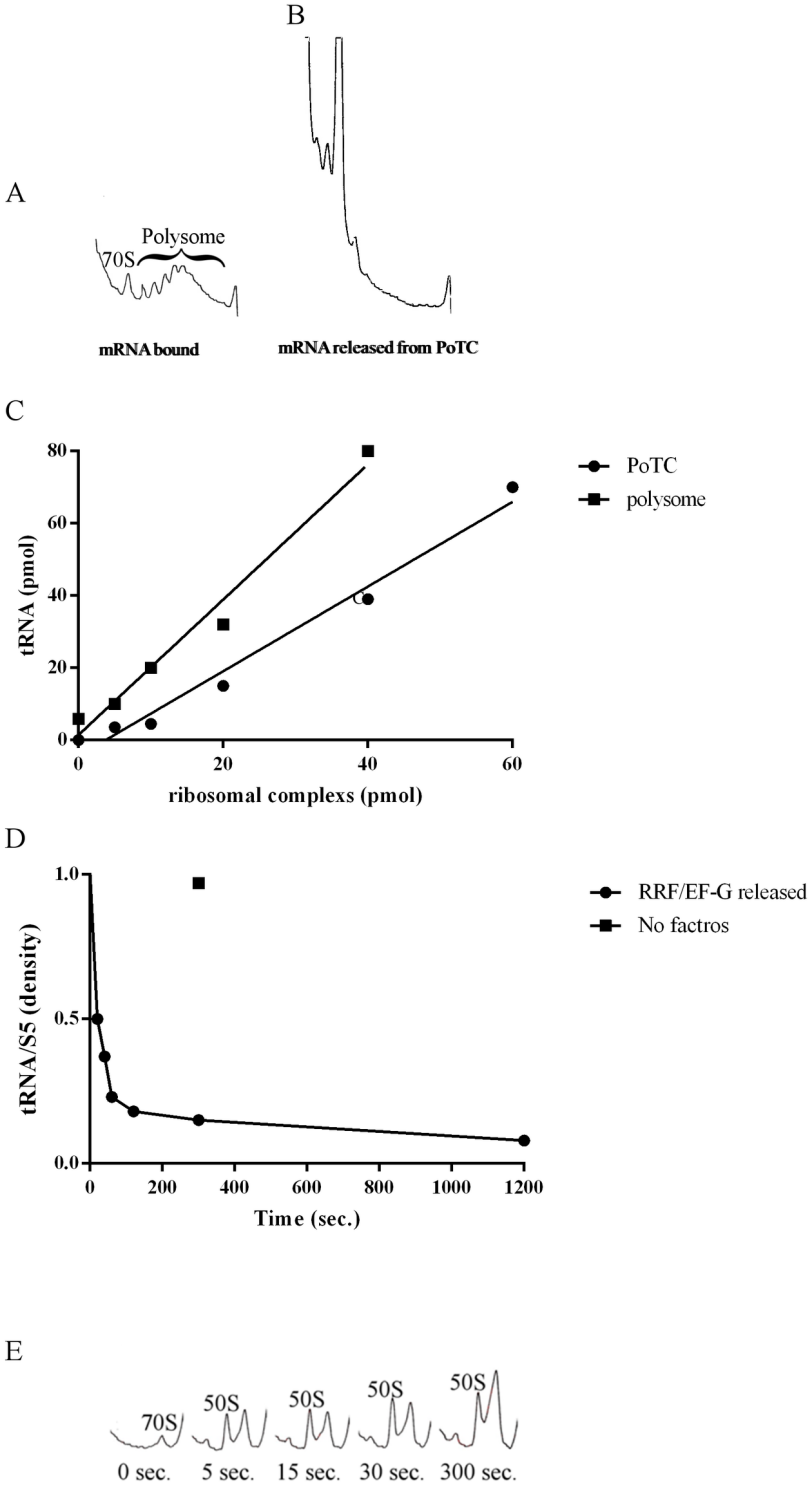
Biochemical evidence for mRNA release from the PoTC. PoTC was incubated in buffer R2 at 30°C for 20 min without (A) or with (B) 5 μM of RRF and EF-G and 500 μM GTP. The reaction mixture was subjected to 15–35% SDGC at 47.6 k rpm for 40 min in rotor SW 50.1, and the sedimentation profile was obtained by ISCO. Sedimentation was from left to right. Ribosomal particles and polysome are indicated in the figure. (C) Total tRNA in polysomes or the PoTC (50 pmoles) was released by RRF and EF-G (2.5 μM each) at 30°C for 10 min (squares, and circles, respectively). The released tRNA was aminoacylated with [^14^C]-amino acid mixture as described in Materials and Methods. The [^14^C]-aminoacyl-tRNA radioactivity was converted to pmoles as described in the supplement. The amount of tRNA thus calculated was plotted against the amount of ribosomal complex (x-axis). (D) The densities of RNA bands (see S3 Fig) were measured and used to calculate the ratio tRNA/5S rRNA and plotted against the incubation period with EF-G and RRF. (E) Sedimentation analysis performed as in Materials and Methods.

Images of RRF reaction products derived from the PoTC after the reaction by RRF, EF-G and GTP were analyzed by cryo-EM (Fig 5C). Due to the ionic conditions (8.2 mM Mg^2+^) in which the disassembly of the PoTC was performed, the ribosomes expected are 70S particles rather than subunits. In fact, most of the ribosomes were 70S ribosomes. From the data presented in Fig 6E (to be discussed in a later section), we believe that these 70S ribosomes were reassociated. It is important to note that the reconstructed cryo-EM structure of the 70S particles (76% of the total particles was used, see S2B Fig) did not show any density attributed to tRNA or mRNA (Fig 5C), even at the lower contour level visualization. The rest of the particles were converged into the distorted 70S ribosome structure at low-resolution, therefore, it is difficult to identify whether tRNA and mRNA exit on this 70S or not. However, by comparing to PoTC dataset the population of particles which has mRNA decreased (S2 Fig). Therefore, this result indicates that bound tRNA and mRNA are released from the PoTC by the reaction with RRF, EF-G and GTP, in support of our preceding papers [1, 14]. This result is in contrast to other publications where very short ORFs were used [9–11].

### Biochemical characterization of the products of the RRF/EF-G reaction on the PoTC

Biochemical evidence that RRF releases mRNA and tRNA from the PoTC and splits subunits is shown in Fig 6A through 6E. In the experiment shown in Fig 6A and 6B, we demonstrated that the release of mRNA from the PoTC occurs. Fig 6A indicates the sedimentation pattern of the PoTC as polysomes before the disassembly reaction. The fact that the ribosomes mostly sediment as polysomes indicates that the mRNA is present in the ribosomes of the PoTC. In contrast, after the RRF reaction (Fig 6B), practically no polysomes were observed, and most of the ribosomes were converted into monosomes. This result clearly indicates that mRNA was released from the PoTC. In the data shown in Fig 6C (circles), we demonstrated that each ribosome of the PoTC releases one tRNA during the disassembly reaction by RRF and EF-G. In contrast, we see that approximately two tRNAs were released from one ribosome of the crude-PoTC (squares). Cryo-EM confirmed this finding, as already discussed regarding Fig 2. It is therefore important to study the release of tRNA from the PoTC and not from the crude-PoTC as in our preceding publications [25]. In the experiment shown in Fig 6D and S3A and S3B Fig, the time course of tRNA release from the PoTC is indicated. It is clear from these data that within 100 seconds after the onset of the reaction, most (80%) of the bound tRNA is released. Moreover, in S3A and S3B Fig, we demonstrate that no tRNA remains after the disassembly reaction by RRF and EF-G.

In the original studies, we never observed the splitting of ribosomes as a result of the RRF/EF-G reaction [1, 26], because the reaction was conducted at 8.2 mM Mg^2+^. However, later studies using a limited amount of subunits translating short ORFs with the SD sequence revealed that the recycling reaction results in splitting of the subunits [9]. This result was possible even though the ionic condition was favorable for subunit association because split subunits were secured at the initiation site due to the strong SD sequence. Later studies all confirmed that the RRF/EF-G reaction indeed resulted in the splitting of the ribosome [10–12]. In our previous studies showing the splitting of the ribosomes, the crude-PoTC was used in the presence of IF3 (initiation factor 3) to prevent the re-association of split subunits. In this section, we examined whether subunit splitting occurred or not using the PoTC in the absence of IF3 at relatively low Mg^2+^ (4 mM) and/or polyamine concentration. In the experiment described in Fig 6E, we show that the recycling reaction indeed splits the ribosome into subunits. Note that the increase in 50S subunits ceased 30 seconds after the onset of the reaction, suggesting that the subunits quickly re-associate into 70S ribosomes even at 4 mM Mg^2+^. In contrast, the amount of 70S ribosomes kept increasing until the end of the reaction. It is noted that the 30S subunit peak does not increase after 5 seconds. However, it is difficult to measure the amount of 30S under the conditions of Fig 6E due to the interference of UV absorbing material from the top of the gradient. We conclude that as a result of the RRF/EF-G reaction, subunits are formed but quickly converted to 70S ribosomes. This is the reason why we observed mostly 70S ribosomes in the cryo-EM studies (Fig 5B).

### IF3 does not directly participate in the recycling reaction catalyzed by RRF and EF-G

Although, in our original studies, we did not observe any role of IF3 in the recycling reaction *per se* [1], Karimi *et al*. implicated IF3 as a functional part of the disassembly reaction. They proposed that IF3 must play a role in releasing deacylated-tRNA based on data obtained from a separately prepared 30S/mRNA/tRNA complex [9]. However, Zavialov *et al*. later indicated that IF3 has no effect on the release of tRNA from the PoTC (Fig 4C of [11]). Moreover, there was no clear evidence that tRNA release was absolutely dependent on IF3 (Fig 4 in [10]). In 1971, Gualerzi *et al*. reported that IF3 releases aminoacyl-tRNA from the 30S/mRNA complex [27]. In contrast, using sonicated polysomes as a substrate for RRF and EF-G, the involvement of IF3 in mRNA release during the recycling reaction was indicated by Singh *et al*. (Fig 1B of [28]).

**Fig 7:**
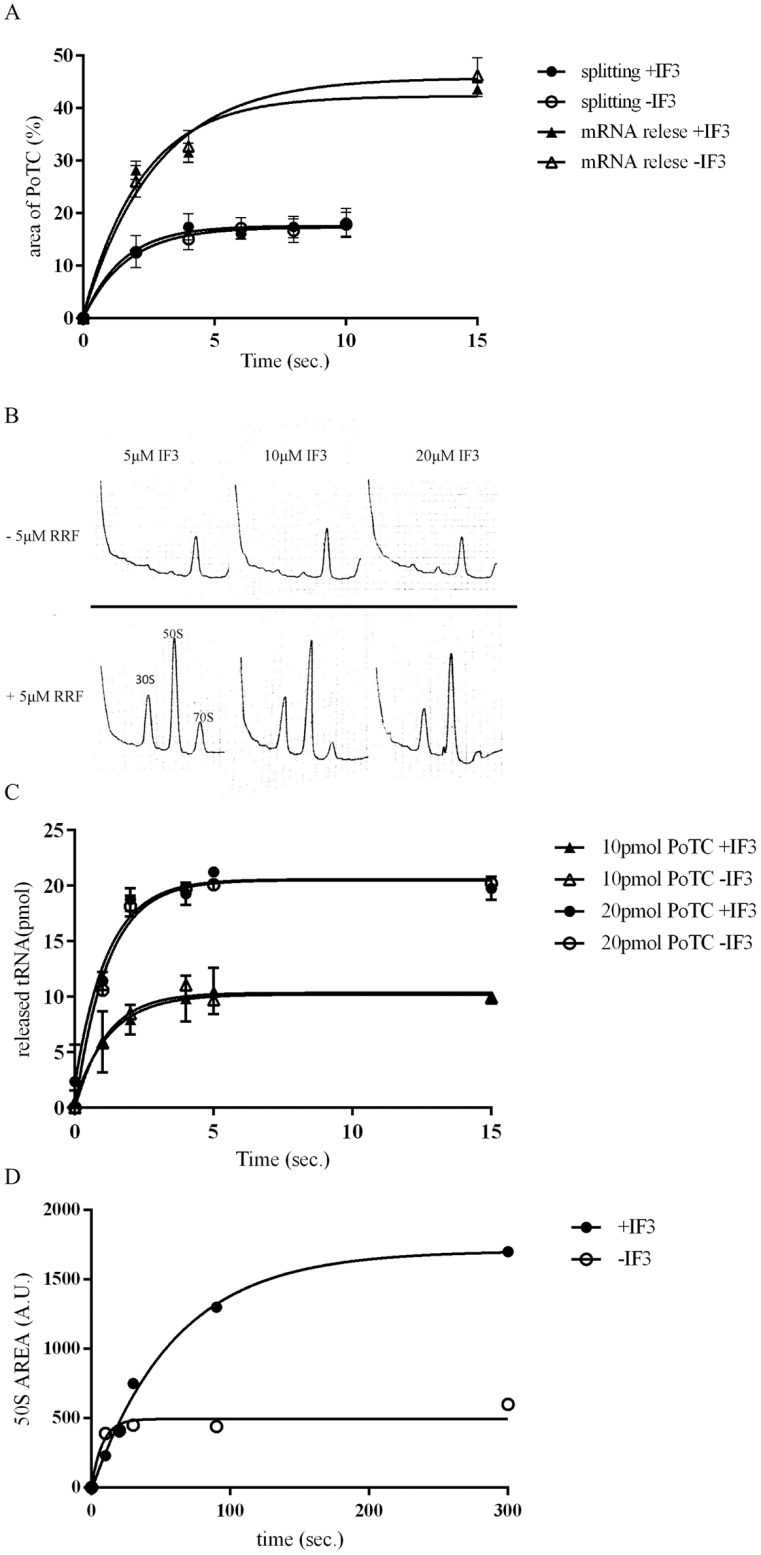
Lack of IF3 effects during disassembly of the PoTC by RRF and EF-G. (A) The release of mRNA and splitting were measured as described in Materials and Methods. When IF3 was present, the final concentration was 4.5 μM. The reaction was performed in buffer R2. Y axis for mRNA release represents the percentage of lost area of PoTC (polysome area) after the reaction. Y axis for splitting indicates the percentage of 50S area compared to the total area of PoTC at zero time. The areas were measured using imageJ (NIH). Depending on the preparation of PoTC, the activity varies. (B) IF3 alone does not split the ribosome into subunits: 0.05 μM of the PoTC, 5 μM of EF-G, 500 μM of GTP and increasing concentrations IF3 (as indicated in the figure) were mixed in the presence or absence of 5 μM of RRF in buffer R2 and incubated for 3 minutes at 30°C. Final volume of the reaction was 60 μL. The reaction was stopped with 100 μL of ice cold buffer R1 and loaded on a sucrose gradient of 15–35% in buffer R1. The gradients were spun for 3 hours and 30 min using an SW50 rotor at 40,000 RPM and analyzed using a ISCO UA-6 spectrophotometer at 254 nm. Sedimentation was from left to right. The sedimentation positions of the 50S, 30S and 70S ribosomal particles are indicated in the figure. IF3 alone was unable to split the PoTC into subunits even at high concentration (20 μM), whereas when RRF was present in the reaction mixture, the 50S and 30S subunits were easily detectable, indicating the splitting of the PoTC. (C) IF3 is not needed for tRNA release. The release of tRNA was measured as described in Materials and Methods. When IF3 was present, the final concentration was 4.5 μM. (D) IF3 does not stimulate the initial rate of ribosome splitting but is required for preventing the split subunits from re-associating. Splitting of ribosome was measured as described in Materials and Methods. When present, IF3 was 4.5 μM. The sedimentation pattern as shown in S4 Fig was obtained, and the area of the 50S peak was measured and plotted against incubation time.

To settle the confusion described above, we examined the possible involvement of IF3 in the disassembly of the PoTC reaction by RRF. In the experiments described in Fig 7A, the initial rate of mRNA release as well as ribosome splitting in the presence and absence of IF3 were examined. It is clear from Fig 7A that IF3 does not have any direct effect on mRNA release or ribosome splitting, measured by the decrease in polysome area (triangles) or increase in 50S subunits (circles). These data are consistent with the concept that IF3 does not actively participate in the splitting reaction. Direct evidence is also shown in Fig 7B. In this experiment, IF3 was always present in the reaction mixture, but in the upper panel, RRF was omitted. In the lower panel, RRF was included in the reaction mixture. It is clear that for splitting to occur, RRF is essential, and IF3 alone does not split 70S ribosome into subunits. The only function of IF3 is to avoid the re-association of split subunits by RRF.

Fig 7C shows that the initial rate of tRNA release was not influenced by IF3. In Fig 7D, the time course of ribosome splitting in the presence or absence of IF3 is shown. It is clear that IF3 does not influence the initial rate of splitting up to 25 seconds after the onset of the reaction. The effect of IF3 becomes obvious after the initial period of incubation. The amount of 50S subunit does not increase after 25 seconds in the absence of IF3, suggesting that subunits split by RRF and EF-G re-associate to form 70S ribosome. On the other hand, in the presence of IF3, the 50S subunits kept increasing until it reached a plateau after 300 seconds. This result means that the only involvement of IF3 in RRF/EF-G dependent ribosome recycling is to prevent the split ribosome from re-associating.

### Order of events during PoTC disassembly

Using a short ORF with the Shine-Dalgarno sequence, Karimi *et al*. proposed that the 50S subunit is dissociated from the PoTC by RRF and EF-G followed by the release of tRNA by IF3, but mRNA remains on the 30S subunit (Fig 6 of [9]). On the other hand, the Wintermeyer group proposed that the splitting of 70S subunits by RRF and EF-G occurs first, leaving the 30S/tRNA/mRNA complex, which is eventually disassembled into each component by IF3 (Fig 6 of [10] and Fig 5 of [19]). With crude-PoTC, we proposed that mRNA/tRNA are released from the ribosome by RRF and EF-G. At that time, we were not aware of the ribosome splitting reaction by RRF and EF-G [8].

**Fig 8:**
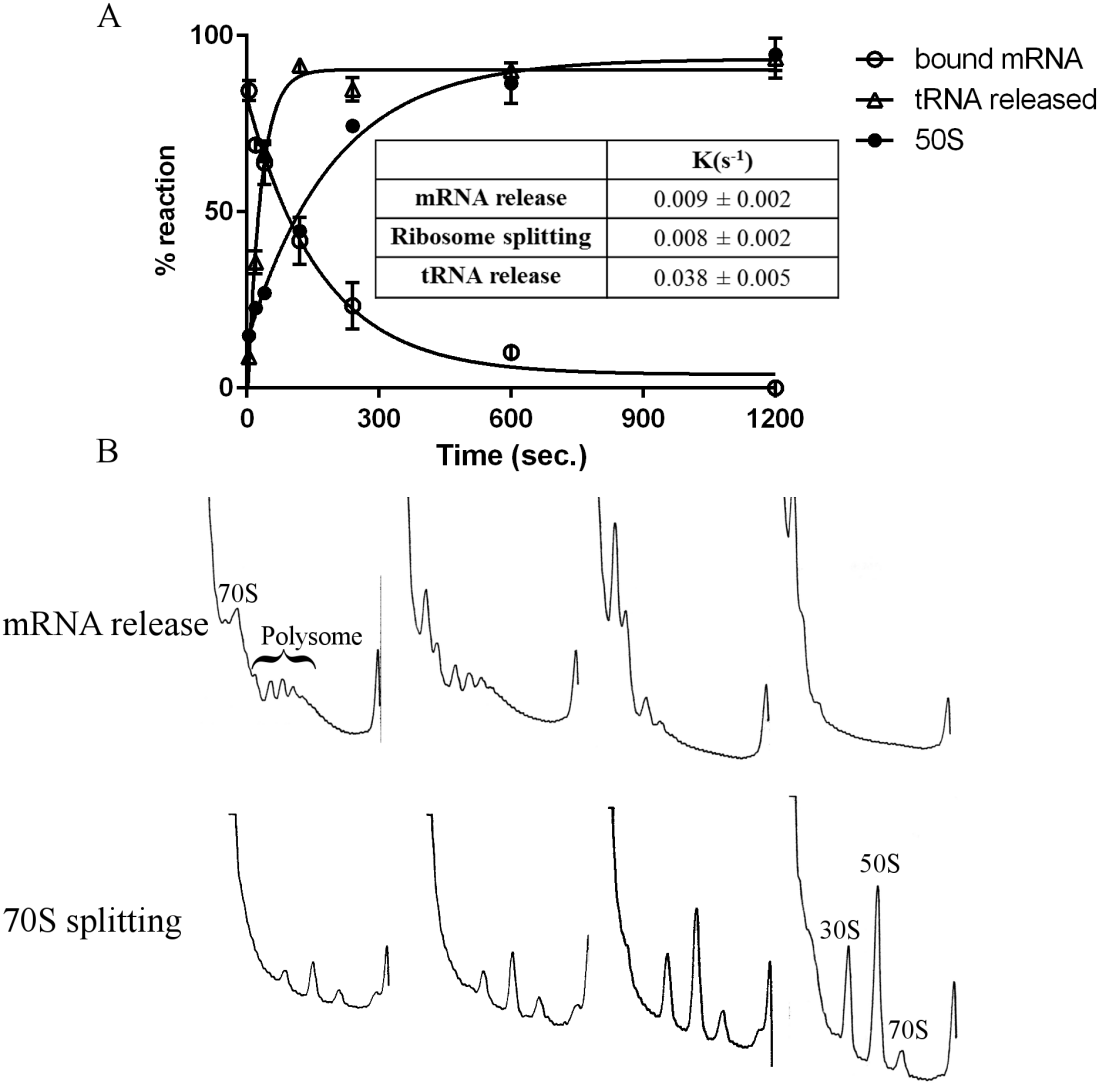
tRNA is released first followed by mRNA release and ribosome splitting during the recycling reaction. PoTC (0.05 μM) was incubated with RRF (5 μM), EF-G (5 μM) and GTP (500 μM) in the presence of IF3 (4.5 μM) in buffer R1 at 30°C. To stop the reaction at various times, a portion (13 pmol PoTC) of the reaction mixture was taken and placed on ice. The released tRNA (open triangles) from 10 pmol of PoTC was measured as described in Materials and Methods. The rest of the PoTC (3 pmol) was analyzed for mRNA release by ISCO (open circles) and splitting of 70S ribosomes (closed circles). (A) Time course of the disassembly of the PoTC. 100% of reaction, representing complete disassembly, was obtained from exposure of the PoTC to 1 mM Mg^2+^. Inset table show the rate constants of the three reactions (mRNA, tRNA release and splitting of ribosomes). (B) Raw data showing the sedimentation pattern of polysomes and 50S subunits. Sedimentation was from left to right.

In the experiment described in Fig 8A and 8B, a master reaction mixture containing all the components, including RRF, EF-G and IF3, but not the PoTC, was prepared at time zero. The reaction was started by the addition of the PoTC, aliquots were taken at various times, and the reaction was stopped by placing the aliquot on ice. The aliquots were divided into three parts for the assay of mRNA release, tRNA release, and ribosome splitting. In S1 Table, we show that placing the reaction mixture on ice effectively stops the reaction. For example, after 15 minutes incubation on ice, no increase in tRNA release was observed. Although we established in the previous chapter that IF3 is not involved in the disassembly reaction *per se* we, added this factor because the split subunits will be reassociated under the buffer conditions used for the reaction, as already shown in Fig 7D. It is clear from Fig 8 that k (rate constant) of the tRNA release was largest, followed by mRNA release and ribosome splitting, in this order. However, considering the limits of the assay, we must conclude that the rates of ribosome splitting and mRNA release were approximately similar. The 100% value was based on the amount of tRNA released from the PoTC in the presence of RRF and EF-G, GTP in buffer L. On the other hand, the release of mRNA was based on the area of SDGC of the PoTC without any factor or incubation. The degree of subunit formation was based on the amount of 50S subunits upon dissociation of the PoTC in buffer L in the presence of RRF/EF-G/GTP after 20 minutes of incubation. These data are shown in Fig 8B.

**Fig 9:**
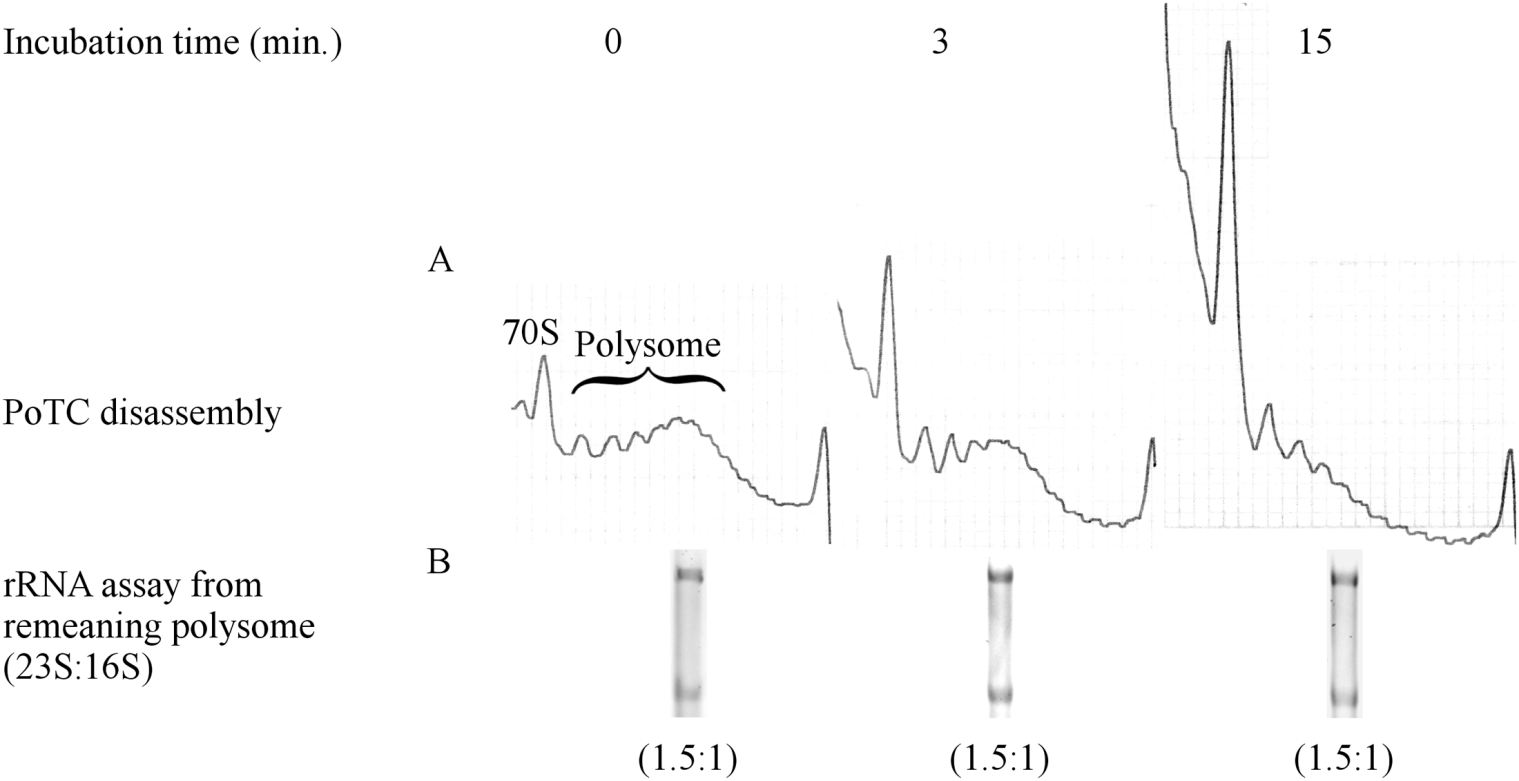
Lack of Poly-30S formation during PoTC disassembly. (A) The PoTC was incubated with RRF and EF-G/GTP as in Fig 8, except that the mixture did not contain IF3, and the reaction was performed in buffer R1 and stopped by viomycin (200 μM final concentration) at 0, 3, and 15 min after the onset of the RRF reaction. After stopping the reaction, 3 pmol of PoTC was subjected to 15–35% SDGC. Sedimentation was from left to right. (B) Total RNA was extracted from the polysome in (A), and the amounts of 23S rRNA and 16S rRNA were determined using UREA-PAGE. The numbers under the gel indicate the ratio of 16S rRNA/23S rRNA in the polysome fraction.

The conclusion from the preceding section is clearly in contrast with the order of events published by two other laboratories [9–11, 19] as described at the beginning of this section, especially with respect to the timing of splitting. These groups suggest that the 50S subunit leaves the PoTC first, leaving the 30S subunit on the mRNA. Although they do not mention the fate of the 30S subunit/mRNA complex clearly, the implication was that mRNA will dissipate from the 30S subunit spontaneously. We wished to examine this possibility in our system by checking the presence of the 30S/mRNA complex during disassembly in our system. For this purpose, in the experiment shown in Fig 9, we isolated polysomes at various times after the onset of the reaction. The idea is that if such an intermediate of 30S/mRNA complex exists, one would expect an increase in the ratio of 16S RNA to 23S RNA in the remaining polysome. To test this possibility, ribosomal RNA was isolated from the PoTC in the polysome at various times after the reaction. The ratio of 23S RNA/16S RNA (1.5, based on the density of each RNA) remained unchanged throughout the incubation period. This result strongly supports that no splitting occurs prior to the release of mRNA from the PoTC. We consider the possibility that the 30S/mRNA complex escapes detection due to its possible lability. However, this behavior is unlikely due to the high Mg^2+^ during the SDGC.

**Fig 10:**
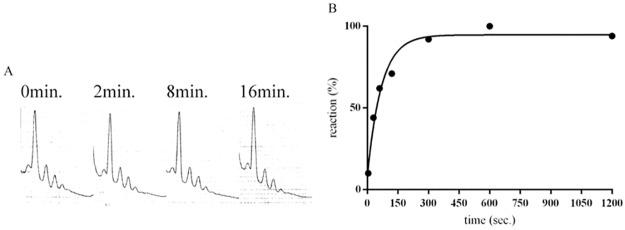
RRF/EF-G do not release polyU from polyU/ribosome/tRNA^phe^ complex. (A) Lack of release of polyU from polyU/ribosome/tRNA^phe^ complex. The reaction mixture (1 ml) for the formation of the complex contained 8 mg of ribosome, 4.1 mg of tRNA^phe^, 0.5 mg of polyU, 140 mM Tris-HCl pH 7.6, 60 mM NH_4_Cl, 6 mM β-mercaptoethanol and 6 mM Mg(OAC)_2_. When prepared in the presence of 6 mM Mg(OAC)_2_, only one tRNA is bound to the P site of the ribosome [29]. The mRNA release was analyzed as described in the Materials and Methods section. (B) Time course of the release of tRNA^phe^ from polyU/ribosome/tRNA^phe^ complex prepared as described in (A). The tRNA release was measured as described in the Materials and methods.

From the studies discussed above, it appears that the behavior of ribosomes changes depending on the mRNA sequence of the PoTC. To confirm this idea, we studied the disassembly of PoTC prepared with polyU and ribosomes. Since the complex was made under ionic conditions allowing only one tRNA per ribosome to bind [29], we assumed that the configuration was similar to the PoTC. Fig 10A clearly shows no change in the polysome area, meaning that the mRNA (polyU) was not released by RRF/EF-G. On the other hand, the release of tRNA^phe^ from the polyU-PoTC occurred at a similar rate to the release of tRNA from the PoTC (Fig 10B). It is well-known that binding of poly U to ribosome is extremely efficient even without a SD sequence suggesting a very high affinity for the ribosome, resulting in extremely high efficiency for incorporation of phenylalanine into polypeptide (poly-phenylalanine) [30]. It is therefore understandable that ribosome will not be released from polyU in a similar fashion that ribosomes will not be released from short ORF with SD sequence.

## Discussion

### Characterization of the substrate

In this paper, we prepared PoTCs that we believe to be as close as possible to the naturally occurring PoTC and attempted to settle the controversy regarding the number and position of tRNAs on the PoTC. The substrate used is similar to what has been reported recently based on crystallographic analysis [20], except that their complex has tRNA exclusively at the P/P site, while the major population (34% of the total) of our PoTC has one tRNA in P/E hybrid state with only 17.4% in this configuration (S2 Fig). One additional important difference is that theirs has a Shine-Dalgarno sequence but ours does not. Furthermore, they prepared their complex by simply mixing mRNA and tRNA^phe^ and ribosome without any enzymes under conditions favorable for the formation of ribosomal complexes with RNAs. Differences between the crystal structure of RRF on the ribosome and structures obtained by other means such as cryo-EM [31] or hydroxyl radical probing [32] were noticed from the early days of structure determination of RRF on the ribosome. In the current study, the majority of tRNA is at the P/E site (Fig 3A and 3B). Since the complex in [20] does not include the termination codon at the A-site, it is similar to ours in this sense. Our PoTC was derived from polysomes, and most if not all of the ribosomes contain triplets other than the termination codon at the A-site. Therefore, release factors RF1, 2 and 3 do not function except at a very slow rate [33]. We therefore removed the nascent peptide chain using puromycin. In our prior studies, to overcome the problem that the substrate does not have the real termination codon at the A-site, we constructed the PoTC, which occurs during the infection of *E*. *coli* with R17 phage having the amber mutation on the seventh codon of the coat cistron [34]. This complex is the only natural PoTC used thus far for the reaction of RRF and EF-G. We confirmed every finding obtained with our model PoTC with this natural system. In addition, we were able to show that in the absence of RRF, the ribosome will start translating from the 8^th^ codon to continue the translation of the coat protein [35]. This was termed “unscheduled translation” due to the loss of RRF. In an equivalent situation with the yeast recycling system, we show that removing the peptidyl group using puromycin or normal termination factors did not cause a significant difference in the behavior of the ribosome [36].

### The release of mRNA

Now that the substrate is reasonably characterized, we can discuss the disassembly reaction, which consists of three separate and closely related events: the release of mRNA, the release of tRNA and the splitting of the ribosomes of the PoTC. An important difference between our conclusion and the conclusions derived by others using defined short mRNAs and strong SD sequences [9–11, 15, 16] is whether the mRNA is released from the PoTC by RRF and EF-G. This difference is important because it bears on the very basic function of the ribosome recycling factor. Ever since we discovered RRF [17], we maintained that one of the important functions of this factor is to release mRNA from the PoTC. Therefore, the original name of RRF stands for ribosome releasing factor, meaning the factor that releases mRNA from the PoTC. We believe that this reason is why RRF is essential for life, as the ribosome must initiate a new round of translation as soon as it completes one. This process requires the ribosome to be released from the mRNA when translation is completed. Ribosomes then bind to the new mRNA, fulfilling the function of RRF, to recycle the spent ribosomes. Our PoTC was created by artificially releasing nascent peptide from peptidyl tRNA. Since the majority of the ribosomes in the polysomes are not within a few nucleotides from the initiation site, we regard them as free of the SD sequence. A search for the presence of the SD-like sequence used by Pavlov *et al*.[15] in all ORFs of *E*. *coli* resulted in 23 hits, and none of them is within 100 nucleotides of the termination codon of the ORF. Moreover, Li *et al*. [37] studied the occurrence of an SD-like sequence in *E*. *coli* mRNA, and there was no SD-like sequence within 20 nucleotides of the end of the ORF, except for some limited case. This result means that one should not use mRNA with an SD sequence near the PoTC to study the behavior of ribosomes during the natural recycling reaction. The naturally occurring PoTC contains mRNA whose average length is far longer than any mRNA used by other laboratories, which do not show the release of mRNA by RRF and EF-G. In this sense, our PoTC is closer to a natural post-termination complex because it was prepared from growing cells.

Moreover, closer examination of the papers concluding that RRF does not release mRNA revealed data supporting our conclusions that RRF does release mRNA. For example, Fig 5C of [10] illustrates the binding of mRNA with strong SD (fluorescence labeled) upon loss of mRNA without SD sequence. They show that the maximum binding of new mRNA (revealed by increased fluorescence) occurs in the presence of RRF, EF-G, IF1, 2, 3 and fmet-tRNA. We interpret these data to indicate that RRF/EF-G releases the mRNA without SD in preparation for the new mRNA with SD to bind to the released vacant ribosome. In conclusion, to study the true RRF function, one must not use a short ORF with SD sequence.

### The release of tRNA

Regarding the release of tRNA, we agree with the more recent paper from the Ehrenberg group that it is not dependent on IF3 (Fig 4C of [11]). Initiation factors function to bind new mRNA [38] but not to release tRNA or mRNA. Although Peske *et al*. [10] (Fig 4 of their paper) indicate that some tRNA is released by RRF and EF-G and, under their conditions, that IF3 stimulates the reaction, our data clearly indicate that IF3 is not needed and does not stimulate the release of tRNA from the PoTC (Fig 7C). Our conclusion was supported by others (Fig 4C (▲) of [11]).Why then was IF3, of all factors, implicated in the recycling reaction? There is a good reason for this. IF3 has the capacity to release aminoacyl tRNA from the 30S/mRNA complex [27], preventing unwanted protein synthesis induced by accidental complex formation between mRNA and aminoacyl tRNA on the 30S subunits.

### Splitting of 70S ribosomes

Earlier studies in our laboratory [12] revealed that the vacant 70S ribosome can be split by EF-G and RRF. IF3 assists this reaction by preventing the re-association of split subunits into the 70S ribosome, in confirmation of the observations by others [9, 10, 15, 16, 34, 39, 40] that RRF/EF-G indeed splits 70S ribosomes. We emphasize that anti-association activity of IF3 is an old well established observations [39, 40] and we simply confirmed this fact in our system, In this study, we showed that, using PoTC derived from natural polysomes that are not near a SD sequence, RRF and EF-G were able to split the PoTC into subunits even in the absence of IF3 (Fig 7A, open circles). Despite the importance of the splitting reaction by RRF/EF-G, we do not believe it to be the reason RRF is an essential gene in bacteria, as there are other factors that dissociate the ribosomes into subunits [41]. Furthermore, in many cases, the initiation of protein synthesis occurs through 70S ribosomes [42, 43]. In fact, recent evidence indicates that a genetically engineered *E*. *coli* strain that cannot split the 70S ribosome grows with 60% efficiency compared to the wild type [44]. This intriguing result suggests further studies are necessary on the essential function of RRF. Such studies are in progress.

### Order of events

We believe that the order of events is greatly dependent on the mRNA sequence of the PoTC. For example, RRF cannot release polyU bound to the ribosome (Fig 10A). Therefore, everyone is correct in their conclusion as long as they limit it to the mRNA they are using. The misconception that the mRNA is not released by RRF/EF-G originated from the use of mRNA with a short ORF combined with a strong SD sequence. It is well known that the speed of ribosome movement on the mRNA is slowest during the early part of mRNA because it is close to the SD sequence [37]. Furthermore, we showed that the inactivation of RRF *in vivo* caused reading of the untranslated 3’ region of mRNA (unscheduled translation) [45]. This result is an *in vivo* evidence that RRF releases ribosomes from mRNA. In the continuation of our studies using phage GA coat and the lysis gene connected with UAAUG [46], we have evidence that *in vivo* unscheduled translation was completely abolished if the upstream ORF is short with an SD sequence (to be published elsewhere). These data show that due to the strong “pull” of the SD sequence, the ribosome cannot travel away from the ribosome binding sequence.

It should be noted that the order of events of the disassembly of PoTC is closely related to the Mg^2+^ concentration and it is impossible to conduct experiments under the *in vivo* Mg^2+^. PoTC-like complex with strong SD sequence is split well by IF1 and IF3 but not by RRF and EF-G. On the other hand PoTC without SD was split well by RRF and EF-G. It was also noted that ribosome splitting by RRF and EF-G was faster with P-site tRNA. In addition, the kind of tRNA in the P-site change the rate of splitting [15]. Because our PoTC was derived from natural polysome our results would take care of this difference due to the nature of the substrate. Further studies are necessary with natural PoTC without near-by SD sequence. Such a study is in progress and the results will be reported elsewhere.

**Fig 11:**
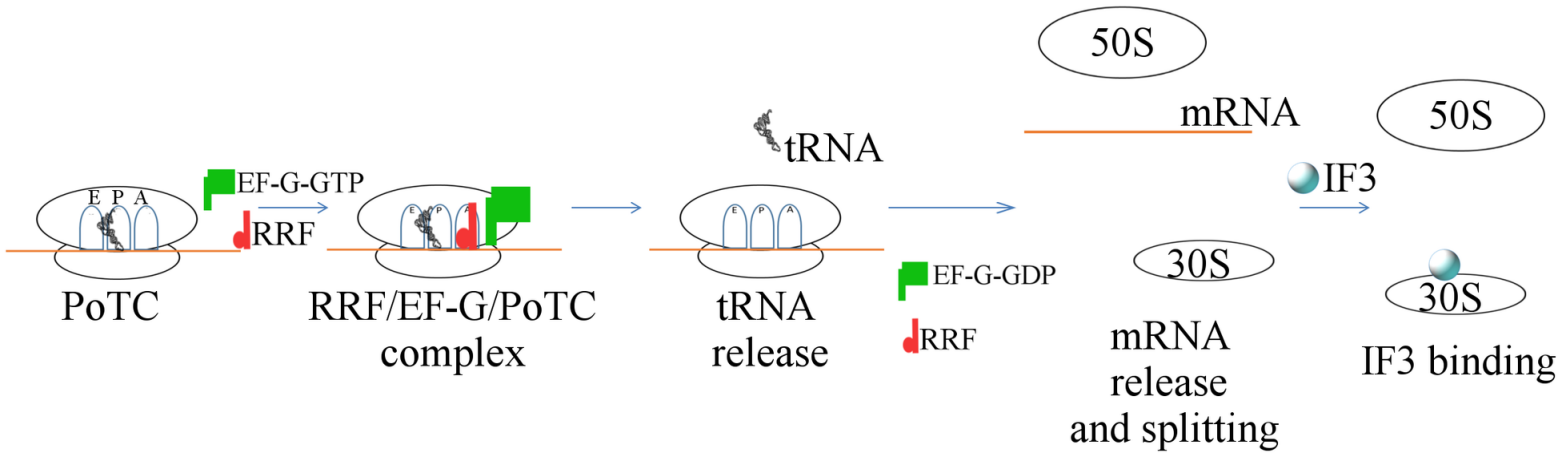
Schematic representation of the order of events with naturally occurring PoTC.

In summary (Fig 11), the order of events during the recycling step is: release of tRNA, followed by almost simultaneous release of mRNA and splitting of the 70S ribosome of PoTC. It should be pointed out that this is the first demonstration of mRNA release in comparison with other events, tRNA release and ribosome splitting in the absence of influence of the SD sequence without the use of inhibitors. Moreover, we showed that recycling of the PoTC by RRF and EF-G is not dependent on IF3, which only functions in recycling as anti-association factor.

## Materials and methods

### Buffers

The buffer contents are as follows: Buffer F: 10 mM Tris-HCl (pH 7.6), 10 mM MgOAc_2_, 50 mM NH_4_Cl, 1 mM DTT; Buffer H1: 50 mM Tris-HCl (pH 7.6), 100 mM NH_4_Cl, 7 mM β-mercaptoethanol; Buffer H2: 20 mM Tris-HCl (pH 7.6), 100 mM NH_4_Cl, 7 mM β-mercaptoethanol; Buffer C: 100 mM Tris-HCl (pH 7.6), 5 mM MgCl_2_, 50 mM KCl, 0.5 mM EDTA; Buffer P: 10 mM Tris-HCl (pH 7.6), 20 mM MgOAc_2_, 50 mM NH_4_Cl, 1 mM DTT; Buffer R1: 10 mM Tris-HCl (pH 7.6), 8 mM MgOAc_2_, 50 mM NH_4_Cl, 1 mM DTT; Buffer R2: 10 mM Tris-HCl (pH 7.6), 4 mM MgOAc_2_, 50 mM NH_4_Cl, 1 mM DTT; Buffer Po: 10 mM Tris-HCl (pH 7.6), 15 mM MgOAc_2_, 50 mM NH_4_Cl, 1 mM DTT; Buffer L: 10 mM Tris-HCl (pH 7.6), 1 mM MgOAc_2_, 50 mM NH_4_Cl, 1 mM DTT.

### Factors

RRF was purified from *E*. *coli* DH5α harboring plasmid pRR2 [4], as previously described [1]. The purified RRF was dialyzed against buffer F and stored at -80°C. EF-G was purified from *E*. *coli* JM83/pECEG [47] based on published methods [48]. The purified EF-G was dialyzed against buffer F and stored at -80°C. His-tagged IF3 was purified from *E*. *coli* XL1-blue containing plasmid expressing His-IF3 (kindly supplied by Dr. T. Ueda) as described in Shimizu *et al*. [49] with slight modifications. Buffer H1 was used for cell lysis and the Ni^2+^ chromatography step. His-IF3 was dialyzed against buffer H2 after elution with imidazole.

### Polysome (crude PoTC) preparation

Polysomes were isolated from *E*. *coli* strain Q13 (CGSC 4947, ATCC 29079) as described in Hirashima and Kaji [1] with the following modification. The crude polysomes (3–4 ml) after lysing protoplasts were run on 6 ml of 15–50% SDGC (Sucrose Density Gradient Centrifugation) on 1.2 ml of 80% sucrose cushion in buffer P at 36.7 k rpm for 4 hrs in a Beckman (Indianapolis) SW41 rotor and then fractionated every 300 μl from the bottom of the tube. Absorption of every fraction was measured at 260 nm, and the polysome profile was examined using an ISCO UA-6 spectrophotometer. The polysomes fractions (fractions containing complexes larger than monosomes) were then collected, and sucrose was removed using Amicon YM-100. To re-isolate polysomes without subunits and 70S ribosomes, if necessary, the above isolated polysomes were further subjected to 15–35% SDGC in buffer P. To determine the amount of polysomes or PoTC, PoTC was subjected to a spectrophotometric assay at 260 nm, where 1 O.D. at 260 nm corresponds to 24 pmol of ribosomes per ml [50].

### PoTC preparation

To make PoTC, 0.1–0.5 μM polysomes was incubated with 5 μM EF-G, 500 μM GTP and 50 μM puromycin in buffer Po for 3 min at 30°C. The PoTC was isolated and washed by Amicon YM-100 (0.5 ml) at 2000 x g with buffer Po.

### tRNA release and measurement by aminoacylation or UREA-PAGE

PoTC (0.1 μM) was incubated with RRF (5 μM), EF-G (5 μM), and GTP (500 μM) in buffer R1. The released tRNA from polysomes (crude PoTC) or the PoTC was measured as described [8]. Briefly, the released tRNA was isolated using a nitrocellulose membrane (pore size 0.45 μm) and aminoacylated with 0.3 μCi of [^14^C]-amino acid mixture (NEC445E, PerkinElmer) by aminoacyl-tRNA synthetase (A3646, Sigma-Aldrich) in 90 μl of buffer C containing 50 units of aminoacyl-tRNA synthetase and 2.5 mM ATP for 12.5 min at 37°C. The cold TCA insoluble radioactivity was regarded as a mixture of [^14^C]-aminoacyl-tRNA formed from tRNA released from the polysomes (crude PoTC) or the PoTC. Based on the radioactivity, the actual amount of tRNA was calculated as described in the supplemental section (S5 Fig). The tRNA amount in the polysomes or PoTC was assayed by UREA-PAGE. Total RNA was extracted from the polysome/tRNA or PoTC/tRNA complexes by phenol/chloroform, followed by incubation with 0.5 mg/ml of proteinase K for 3 hours to overnight, and then precipitated by isopropyl alcohol together with glycogen. The denaturing 12% acrylamide/8 M UREA gel was subjected to 200 V for 30 min. Then, an appropriate amount of RNA equivalent to 5 pmol polysomes or PoTC was loaded, and the gel was run at 90 V until the bromophenol blue reached the bottom of the gel. Transfer RNA and 5S rRNA bands were visualized with transilluminator after staining by SYBR Gold solution (1/10,000 dilution). The positions of the tRNA and 5S rRNA were determined using RNA Century-Plus Markers (Ambion, AM7145). The densities were measured by the ImageJ software (NIH).

### mRNA release assay

PoTC (0.1 μM) was incubated with RRF (5 μM), EF-G (5 μM), and GTP (500 μM) in buffer R1 or R2 at 30°C. The reaction was stopped by cooling in ice. PoTC (3 pmol) was placed on a 15–35% sucrose gradient in buffer R1 or R2 and centrifuged in a Beckman (Indianapolis) SW50.1 rotor at 47.6 K rpm for 40 min at 4°C. The fast sedimenting PoTC, representing the remaining PoTC, was analyzed using an ISCO UA-6 spectrophotometer at 254 nm.

### Subunit splitting analysis by ISCO

Split ribosomal subunits were analyzed by ISCO as described in the mRNA release section except that ultracentrifuge time was prolonged for 160 min to see the ribosomal subunits clearly by removing the remaining PoTC. The reaction mixture without IF3 was stopped by cooling, and then IF3 (4.5 μM) was added to prevent the ribosomal subunits from re-associating.

### Attempt to observe poly-30S during PoTC disassembly by RRF and EF-G/GTP

The disassembly of the PoTC was conducted as above except that samples were taken for sedimentation analysis during the disassembly reaction. Ribosomal RNA in the remaining PoTC was analyzed using 8 M UREA/PAGE to measure the amount of rRNA as follows: the mRNA bound ribosomes were pelleted at 47.6 k rpm for 105 min in a Beckman SW 50.1 rotor. The ribosomal pellet was dissolved in buffer R1, and rRNA was extracted as described in the section on tRNA measurement by UREA-PAGE. The extracted RNA (1 μg) was loaded and subjected to denaturing 12% acrylamide/8 M UREA gel at 90 V until the bromophenol blue reached the bottom of the gel. Ribosomal RNA bands were visualized with transilluminator after staining by SYBR Gold solution (1/10,000 dilution), and the densities corresponding to the 16S and 23S ribosomal RNA were measured by ImageJ software (NIH).

### Cryo-electron microscopy of the PoTC and its disassembly by RRF and EF-G/GTP

For this purpose, 0.1 μM PoTC prepared as described above was incubated with RRF (5 μM), EF-G (5 μM) and 500 μM GTP at 30°C for 20 min in buffer R2. To remove the released tRNA, the reaction mixture was washed three times using the same buffer and passed through Amicon YM-100 filters (3.5 k x g).

Polysomes, PoTC or PoTC treated with RRF and EF-G/GTP described above were diluted to 100 nM with the buffer containing 10 mM Tris-HCl (pH 7.6), 4.5 mM Mg(OAc)_2_, 80 mM NH_4_Cl, 1 mM DTT, and 2% sucrose. These suspensions (4 μl droplet) were placed onto the thin carbon layer-coated Quantifoil R2/2 holy carbon grid (thickness approximately 50 Å) (Quantifoil). The grid was blotted for 5 seconds at 6°C in a 100% humidified chamber to remove the excess solution and immediately plunged into liquid ethane to prepare vitrified specimens using a Vitrobot automated plunging machine (FEI).

To observe specimens embedded in the ice layer using high-contrast images, a JEOL 1230 transmission electron microscope (JEOL) was operated at 120 kV accelerating voltage at liquid nitrogen temperature (77 K, -196°C). Grids were transferred into the electron microscope column using an Oxford cryo-transfer holder (Oxford Instruments). Subsequently, specimens were observed at a magnification of 40,000×, and -3.5 μm defocused images were recorded with a 1 K × 1 K FastScan-F114 CCD camera (TVIPS) (see images shown in Fig 5). To obtain ribosome images with high-resolution structural features, other grids freshly prepared under the same conditions were transferred into a JEM-3100FFC electron microscope (JEOL), which can observe specimens at 300 kV accelerating voltage at liquid helium temperature (4K, -269°C). Specimens were observed at a magnification of 57,500×, and images were recorded on SO-163 films (Kodak). A total of 145 micrographs of the model PoTC were collected at defocuses ranging from –1.0 μm to -3.7 μm, and 96 micrographs of the reaction products of the PoTC were recorded at defocuses ranging from -1.0 μm to -3.1 μm. All micrographs were digitized by scanning with a photo scanner (Z/I imaging) at a scanning resolution of 7 μm.

### Single-particle image analysis of cryo-EM images of the PoTC and its reaction product

Ribosome particle images were semi-automatically picked using the e2boxer.py program implemented in the EMAN2 package [51] with the box size of 300 pixels. Contrast transfer function for each micrograph was estimated using ctffind4 [52]. Particle extraction, 2D classification, 3D classification and subsequent refinement was performed with Relion2[53]. 1) PoTC dataset. 2D classification was performed in the extracted particles. As a result of 2D classification 118,567 particles were selected. Subsequently, 3D classification was performed into seven classes with angler sampling interval at 7.5 degree and global search (S2A Fig). Classified subsets were combined if the overall structure is the same and further refined with relion auto-refine. At this point, 34% of the total particles converged into the rotated PoTC has one tRNA at P/E hybrid state. Refined cryo-EM structure was at the resolution of 8.5 Å (S6 Fig). The other subset, 33% of the total particles converged into the unrotated PoTC has weak P-tRNA and fragmented density in E site (S2A Fig). In order to sort this subset into homogeneous subgroups based on tRNA occupancy, another round of 3D classification with finer angler search (0.9 degree) and local angular search was performed. As a result of the second round of 3D classification, the subset of unrotated PoTC was classified into three subgroups (9.3% of the total, unrotated 70S with P, E-tRNAs and mRNA; 6.2% of the total, unrotated vacant 70S; 17.4% of the total, unrotated PoTC with P-tRNA and mRNA). The unrotated PoTC with P-tRNA and mRNA was further refined and solved at 9.4 Å resolution (S6 Fig). 2) Dataset for the reaction product of PoTC with ribosome recycling reaction. 2D classification was performed in the extracted particles. As a result of 2D classification 73,091 particles were selected. Subsequently, 3D classification was performed into seven classes with angler sampling interval at 7.5 degree and global search (S2B Fig). Classified subsets were combined if the overall structure is the same and further refined with relion auto-refine. As a result, 76% of the total particles converged into the vacant 70S. This dataset was further refined and obtained cryo-EM structure was at the resolution of 9.2 Å (S6 Fig). Even in the lower contour level visualization any densities for tRNA and mRNA were observed. 3) Polysome data. Image processing was performed using SPIDER program [54]. The total images (22,473 images) were sorted into two groups using reference-based classification with rotated and unrotated vacant 70S ribosome structure as references. Unrotated particles (16,719 images) were used for the reconstruction of polysome structure (S6 Fig). Cryo-EM structures were segmented into each component of the complexes for the interpretation of the structures using UCSF chimera program [55].

## Supporting information

S1 Fig Schematic representation of the preparation of PoTC from naturally occurring polysomes. (A) Various forms of ribosomes exist in naturally occurring polysomes. (B) Treating the complexes in (A) with EF-G and GTP results in two possible forms of ribosomes. (C) PoTC was obtained after treatment of (B) with puromycin *in vitro*. One tRNA is released from the E site due to the change of peptidyl tRNA from P/P to P/E site.
(TIF)

S2 Fig Scheme of 3D classification of the PoTC and the reaction products of ribosome recycling reaction. (A) This scheme shows how 3D classifications were performed to obtain cryo-EM structures of PoTCs in rotated and unrotated state. The first round of 3D classification was performed in 118,567 particles selected after 2D classification with the sampling interval at 7.5 degree and global search. 34% of the total particles converged into the rotated PoTC with P/E-tRNA and mRNA. The other subset of particles (33% of the total) converged into unrotated PoTC with P-tRNA, fragmented density in E site and mRNA. The rest of particles (33%) shows distorted 70S structures implying that those were reconstructed from low quality particles. Therefore, those were discarded at this step. In order to classify particles in unrotated state based on the difference of tRNA occupancy, another round of 3D classification was performed with finger angular interval (0.9 degree) and local angular searches. As a result, 9.3% of the total particles converged into 70S ribosome with two tRNAs in P and E sites and mRNA. 6.2% of the total particles converged into vacant 70S. 17.4% of the total particles converged into unrotated PoTC with P-tRNA and mRNA. (B) 3D classification scheme shows the analysis the dataset of the reaction products of the ribosome recycling reaction with PoTC. 3D classification was performed in 73,091 particles selected after 2D classification with the sampling interval at 7.5 degree and global search. 76% of total particles converged into the vacant 70S without any densities for tRNAs and mRNA. The other subset of particles (24% of the total) shows distorted 70S structures implying those are reconstructed form low quality images.
(TIF)

S3 Fig Time course of tRNA release from PoTC by RRF/EF-G. Analysis by UREA-PAGE. (A) The densities of RNA bands from lanes 1 to 8. (B) Densities were measured using ImageJ. The numbers next to each peak indicate the area of the peak. The preparation of RNA from PoTC is described in the material and method section. (C) Absence of tRNA on PoTC after dissociation by exposure to low Mg^2+^. After releasing tRNA from PoTC under low Mg^2+^ (1 mM), the remaining tRNA on PoTC was assayed by UREA-PAGE. Lanes 1 to 4, total RNA from various amounts (pmol) of PoTC was applied; Lane 5, size marker; Lane 6, tRNA^Lys^ (5.0 pmol).
(TIF)

S4 Fig Lack of IF3 effects on splitting and mRNA release of PoTC by RRF and EF-G. Sedimentation is shown from left to right.
(TIF)

S5 Fig Basis of estimation for the molar amount of released tRNA from PoTC. (A) tRNA-Met bound to the purified PoTC was released and aminoacylated with [^35^S]-Methionine as described in the material and method section, except [^35^S]-Methionine (1175 Ci/mmol) was used in place of the [^14^C]-amino acid mixture. (B) Time course of aminoacylation of tRNA released from polysomes. The experimental procedure for the aminoacylation of tRNA released from polysomes is described in the material and method section. Open and closed circles represent 10 pmol and 20 pmol of tRNA released from polysomes, respectively.
(TIF)

S6 Fig Cryo-EM structures and their resolution curves. (A) Cryo-EM structural data shown in this study. From left to right: Polysome, 15.6 Å at FSC 0.5 cutoff; PoTC in rotated state, 8.5 Å at FSC 0.143 cutoff (Gold standard FSC); PoTC in unrotated state, 9.4 Å at FSC 0.143 cutoff (Gold standard FSC); Reassociated 70S, 9.2 Å at FSC 0.143 cutoff (Gold standard FSC). (B) Resolution curves of cryo-EM structures shown in (A).
(TIF)

S1 Table Ice-cold temperature completely stops all RRF reactions, mRNA/tRNA release and ribosomal splitting. The release of tRNA and mRNA from PoTC and ribosome splitting were analyzed at 0 and 15 min after incubation on ice.
(TIF)

S1 File Supporting information. Supplementary information contains control experiments and how to convert CPM into pmoles.
(DOCX)
