# Supplementary figures and images for: Chemical and structural characterization of a model Post-Termination Complex (PoTC) for the ribosome recycling reaction: Evidence for the release of the mRNA by RRF and EF-G
Source: PLoS One. 2017 May 24;12(5):e0177972. doi: 10.1371/journal.pone.0177972 (PMC5443523; doi:10.1371/journal.pone.0177972)

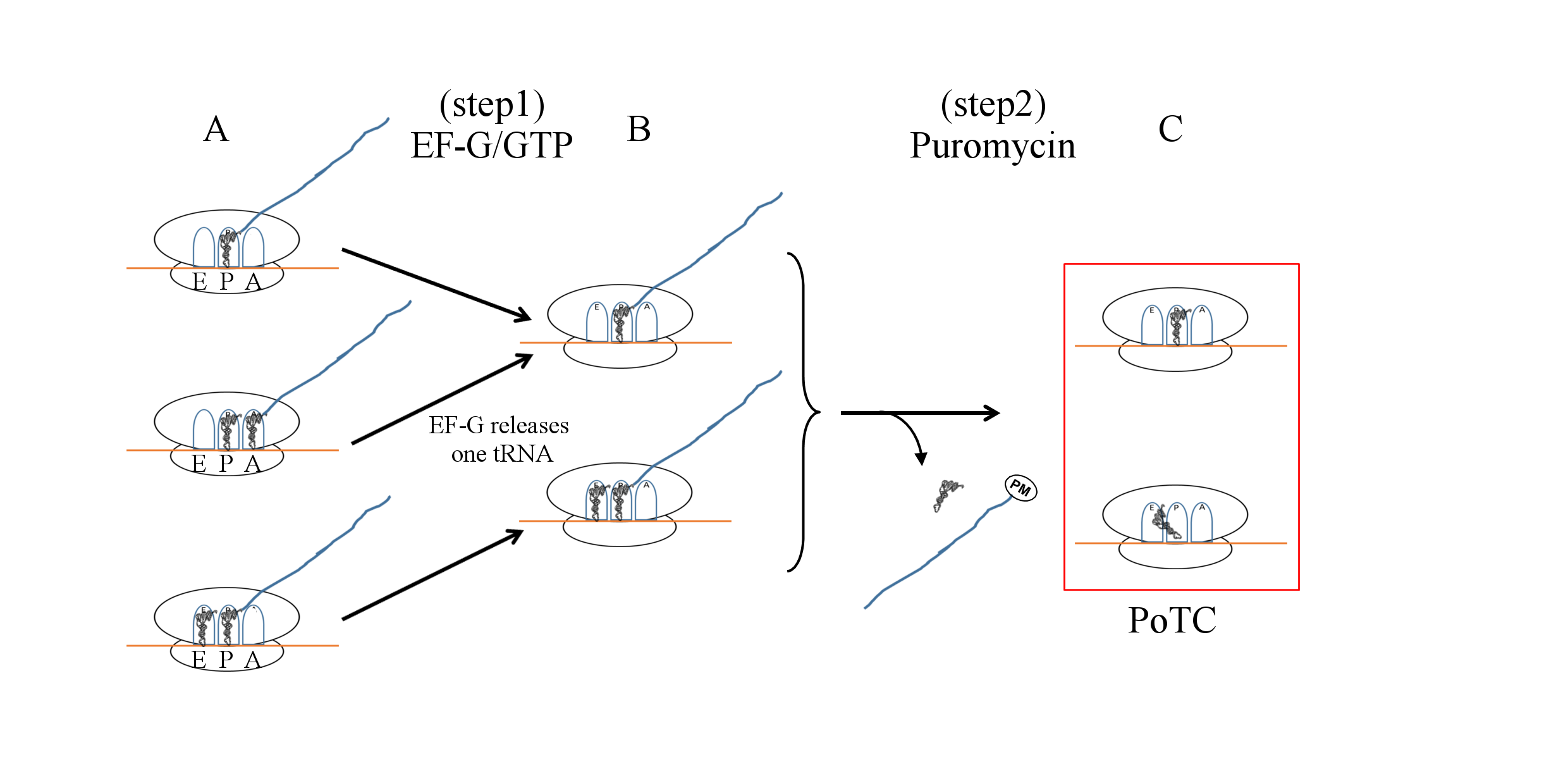

Supplement: S1 Fig — (A) Various forms of ribosomes exist in naturally occurring polysomes. (B) Treating the complexes in (A) with EF-G and GTP results in two possible forms of ribosomes. (C) PoTC was obtained after treatment of (B) with puromycin in vitro. One tRNA is released from the E site due to the change of peptidyl tRNA from P/P to P/E site. (TIF) [file pone.0177972.s001.tif]

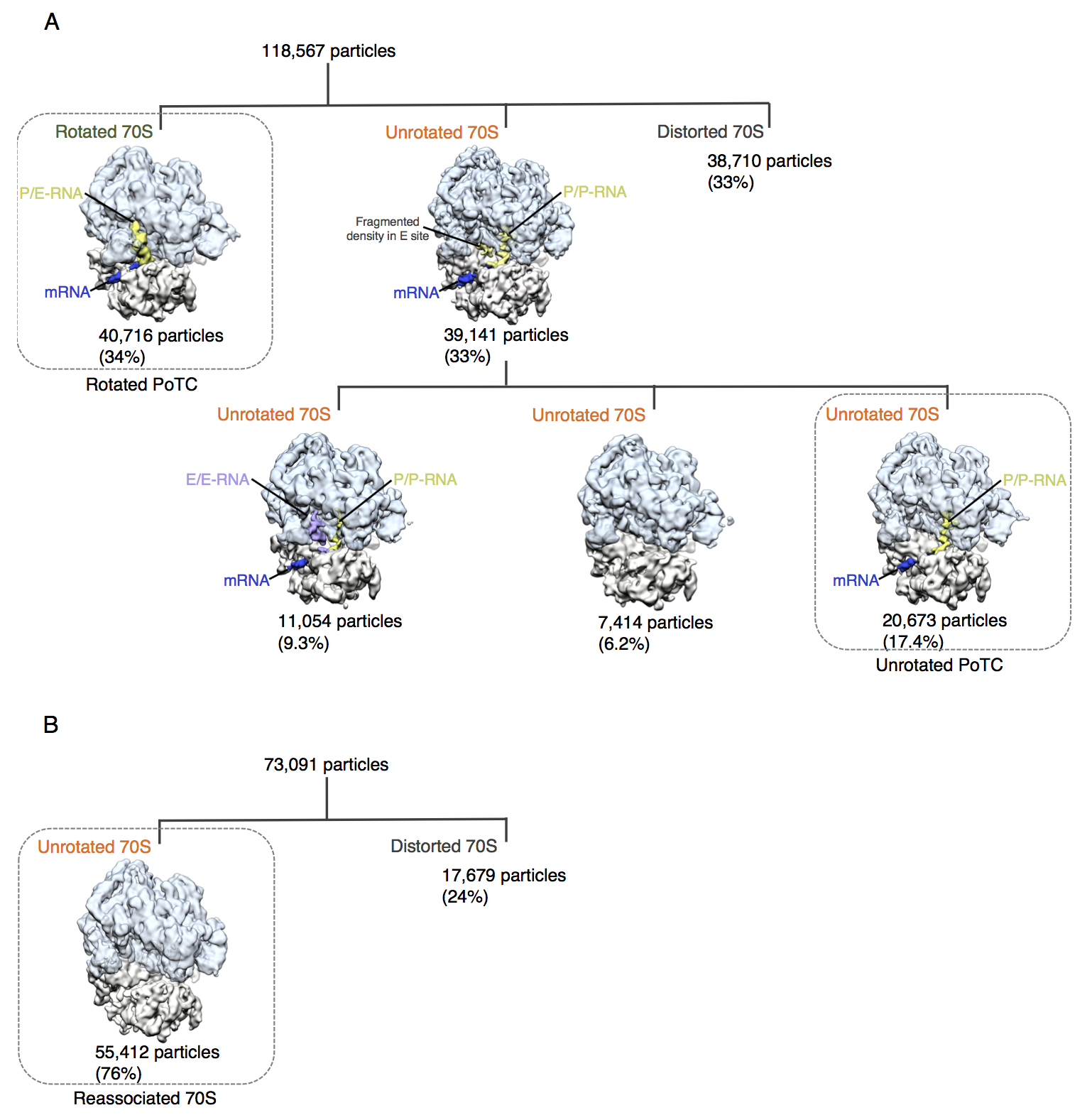

Supplement: S2 Fig — (A) This scheme shows how 3D classifications were performed to obtain cryo-EM structures of PoTCs in rotated and unrotated state. The first round of 3D classification was performed in 118,567 particles selected after 2D classification with the sampling interval at 7.5 degree and global search. 34% of the total particles converged into the rotated PoTC with P/E-tRNA and mRNA. The other subset of particles (33% of the total) converged into unrotated PoTC with P-tRNA, fragmented density in E site and mRNA. The rest of particles (33%) shows distorted 70S structures implying that those were reconstructed from low quality particles. Therefore, those were discarded at this step. In order to classify particles in unrotated state based on the difference of tRNA occupancy, another round of 3D classification was performed with finger angular interval (0.9 degree) and local angular searches. As a result, 9.3% of the total particles converged into 70S ribosome with two tRNAs in P and E sites and mRNA. 6.2% of the total particles converged into vacant 70S. 17.4% of the total particles converged into unrotated PoTC with P-tRNA and mRNA. (B) 3D classification scheme shows the analysis the dataset of the reaction products of the ribosome recycling reaction with PoTC. 3D classification was performed in 73,091 particles selected after 2D classification with the sampling interval at 7.5 degree and global search. 76% of total particles converged into the vacant 70S without any densities for tRNAs and mRNA. The other subset of particles (24% of the total) shows distorted 70S structures implying those are reconstructed form low quality images. (TIF) [file pone.0177972.s002.tif]

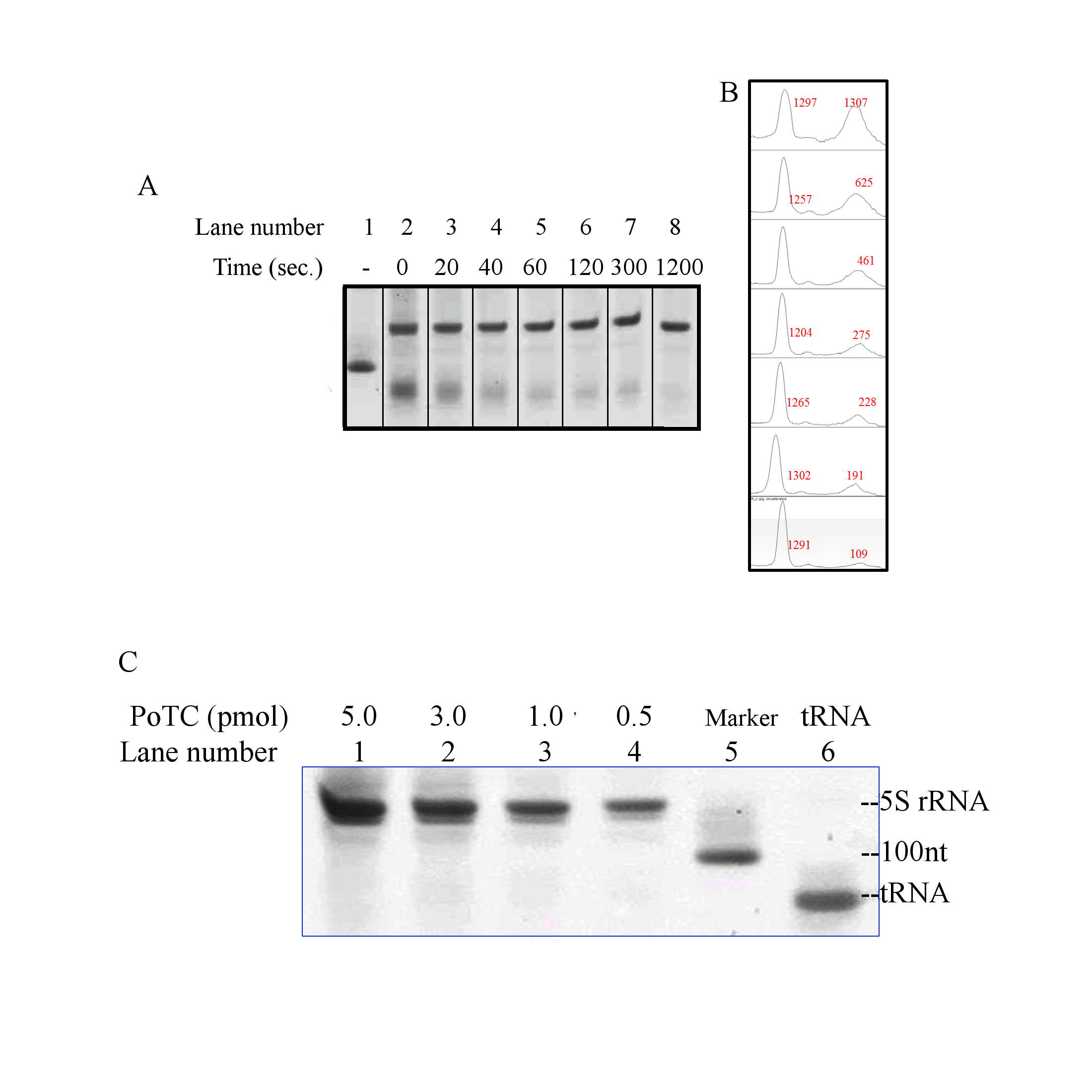

Supplement: S3 Fig — Analysis by UREA-PAGE. (A) The densities of RNA bands from lanes 1 to 8. (B) Densities were measured using ImageJ. The numbers next to each peak indicate the area of the peak. The preparation of RNA from PoTC is described in the material and method section. (C) Absence of tRNA on PoTC after dissociation by exposure to low Mg2+. After releasing tRNA from PoTC under low Mg2+ (1 mM), the remaining tRNA on PoTC was assayed by UREA-PAGE. Lanes 1 to 4, total RNA from various amounts (pmol) of PoTC was applied; Lane 5, size marker; Lane 6, tRNALys (5.0 pmol). (TIF) [file pone.0177972.s003.tif]

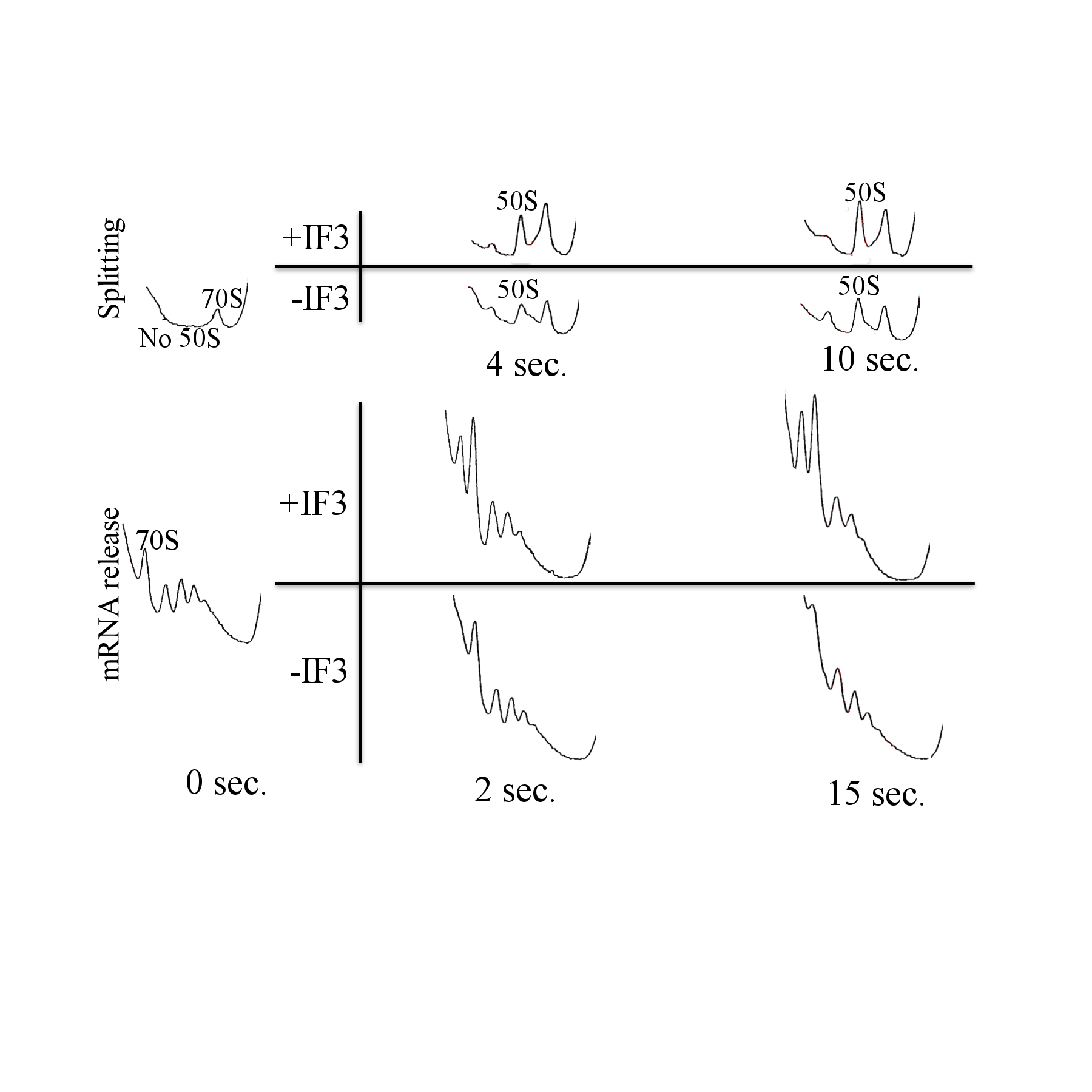

Supplement: S4 Fig — Sedimentation is shown from left to right. (TIF) [file pone.0177972.s004.tif]

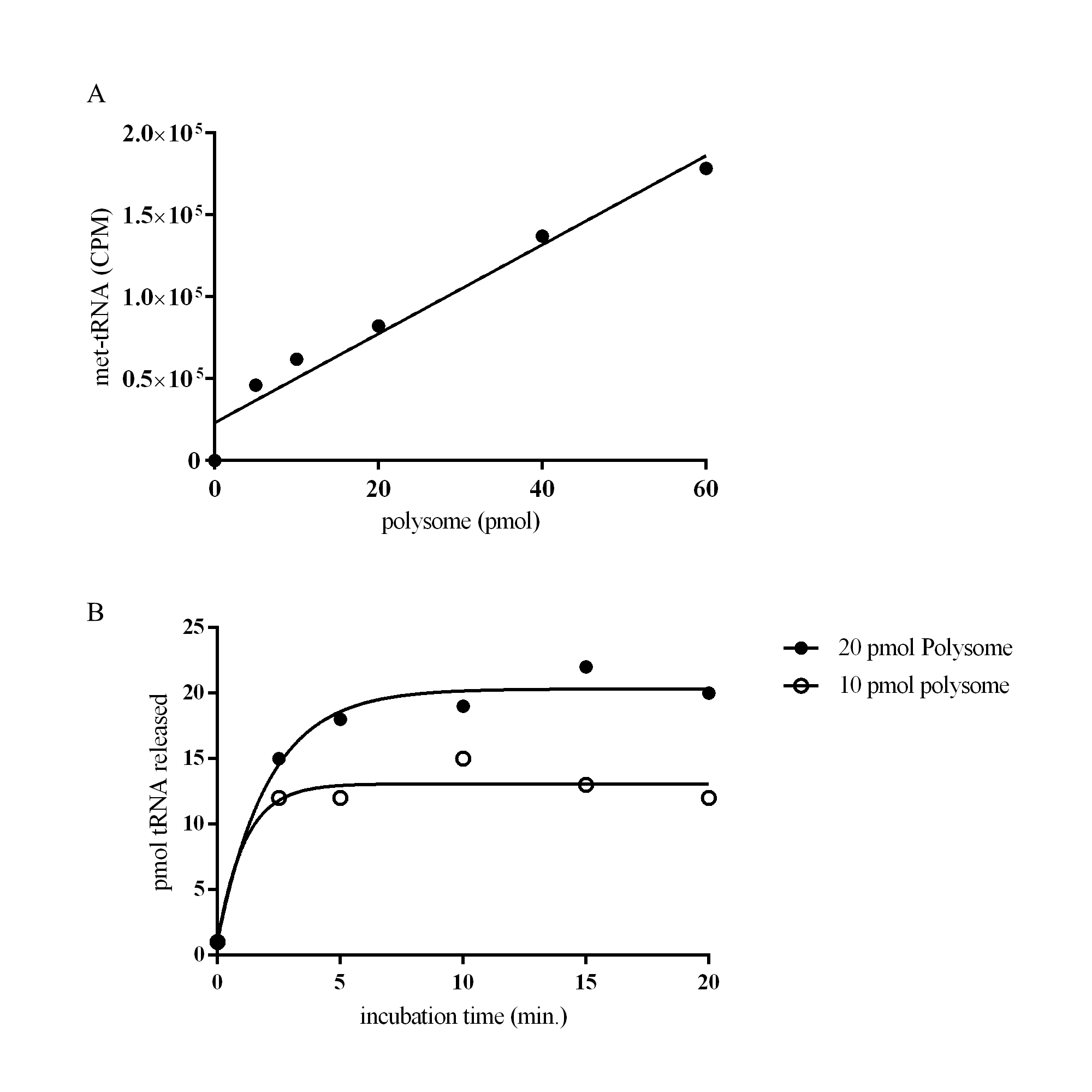

Supplement: S5 Fig — (A) tRNA-Met bound to the purified PoTC was released and aminoacylated with [35S]-Methionine as described in the material and method section, except [35S]-Methionine (1175 Ci/mmol) was used in place of the [14C]-amino acid mixture. (B) Time course of aminoacylation of tRNA released from polysomes. The experimental procedure for the aminoacylation of tRNA released from polysomes is described in the material and method section. Open and closed circles represent 10 pmol and 20 pmol of tRNA released from polysomes, respectively. (TIF) [file pone.0177972.s005.tif]

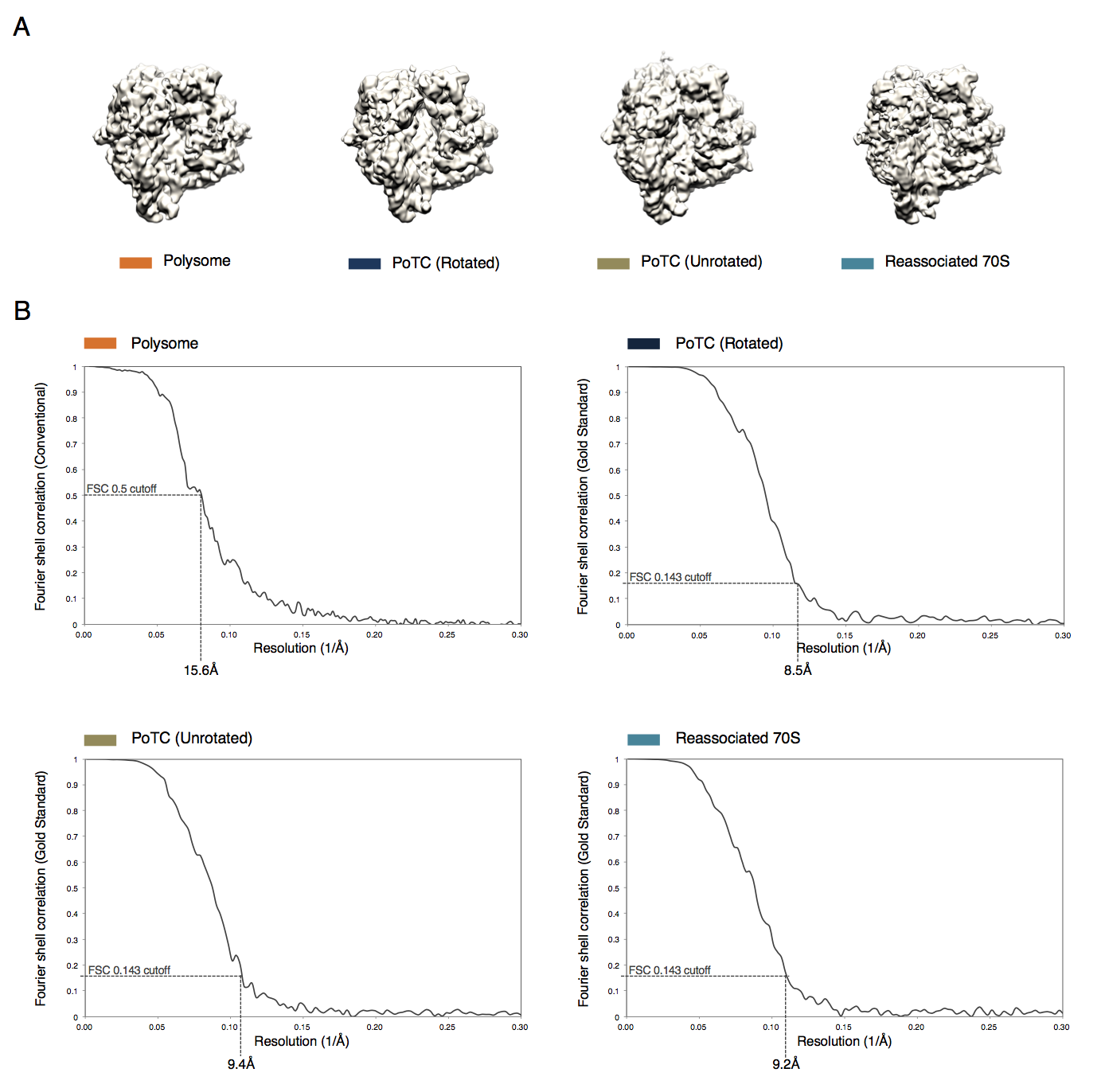

Supplement: S6 Fig — (A) Cryo-EM structural data shown in this study. From left to right: Polysome, 15.6 Å at FSC 0.5 cutoff; PoTC in rotated state, 8.5 Å at FSC 0.143 cutoff (Gold standard FSC); PoTC in unrotated state, 9.4 Å at FSC 0.143 cutoff (Gold standard FSC); Reassociated 70S, 9.2 Å at FSC 0.143 cutoff (Gold standard FSC). (B) Resolution curves of cryo-EM structures shown in (A). (TIF) [file pone.0177972.s006.tif]

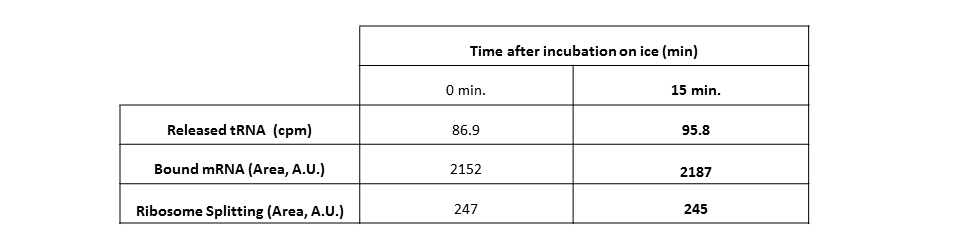

Supplement: S1 Table — The release of tRNA and mRNA from PoTC and ribosome splitting were analyzed at 0 and 15 min after incubation on ice. (TIF) [file pone.0177972.s007.tif]
