# Supplementary material for: Chemical and structural characterization of a model Post-Termination Complex (PoTC) for the ribosome recycling reaction: Evidence for the release of the mRNA by RRF and EF-G
Source: PLoS One. 2017 May 24;12(5):e0177972. doi: 10.1371/journal.pone.0177972 (PMC5443523; doi:10.1371/journal.pone.0177972)
Supplement: S1 File — Supplementary information contains control experiments and how to convert CPM into pmoles. (DOCX) [file pone.0177972.s008.docx]

**Supporting Information**

**Scheme for PoTC preparation**

Pure PoTC were prepared as described in material and method. S1 Fig highlights the main features of the preparation starting from polysome isolated from *E. coli.* [1,2]

**Scheme for 3D classification of the PoTC and the reaction product of the ribosome recycling reaction**

S2 Fig shows 3D classification schemes representing how images of PoTC in rotated and unrotated state (A) and the re-associated 70S (B) were sorted from the datasets.

**UREA-PAGE analysis of the time course of tRNA release from PoTC by RRF/EF-G**

As shown in S3 Fig A and B, most of the bound tRNA (lower band) is released from PoTC within 20 seconds under the experimental conditions. At a low magnesium concentration (S3 Fig C), all of the bound tRNA is non-enzymatically released.

**IF3 is not involved in the disassembly of PoTC by RRF/EF-G (sedimentation data).**

S4 Fig shows the sedimentation pattern of the ribosomal subunits that formed during the initial period of the reaction.

**Estimation of bound tRNA per picomole of crude PoTC:**

We used [^35^S]-Methionine to aminoacylate the mixture of tRNA released from 1 pmol of crude PoTC with RRF and EF-G as described in the material and method, except for the use of [^35^S]-Methionine instead of the [^14^C]-amino acids mixture. Methionine has a frequency of 37.4 per 1000 codons in E. coli (<http://www.kazusa.or.jp/codon/cgi-bin/showcodon.cgi?species=413997>). S5 Fig shows the dose response curve of Methionyl-tRNA formation with increasing amounts of PoTC. It was found that 1 pmol of crude PoTC results in 3278.9 cpm of [^35^S]-Methionyl-tRNA. The scintillation counter efficiency was 0.94 cpm/dpm. By definition, 1nCi is 2220.0 dpm. Therefore, 2220.0 dpm x 0.94 = 2082.7 cpm/nCi. Thus, 3278.9 cpm was equal to 1.57nCi. [^35^S]-Methionine has a specific activity of 1175nCi per pmol. Hence, 1.57 nCi corresponds to 0.0013 pmol of [^35^S]-Methionyl-tRNA per pmol of polysomes. [^35^S]-Methionine was diluted 50 times with unlabeled Methionine, so the final pmol was 0.0013x50 = 0.067 pmol of Methionyl-tRNA per pmol of polysome. This quantity corresponds to 3.74% of the total tRNA, or 0.067 x (100/3.74) =1.791 pmol of total tRNA per pmol of polysomes.

**Ice-cold temperature stops all RRF reactions.**

For the kinetic studies investigating the possible effect of IF3, we used an ice-cold temperature (0° C) to stop the RRF/EF-G reaction. To validate this method and stop all three reactions completely, the reaction mixture was incubated at an ice-cold temperature to determine whether this condition allows any progress of the reaction. As shown in S1 table, a typical reaction mixture was incubated with EF-G/RRF for 15 minutes at an ice-cold temperature. The data suggest that all three reactions stopped completely at an ice-cold temperature.

**Resolution of Cryo-EM structures**

See S6 Fig.

**References**

1. Ishitsuka H, Kuriki Y, Kaji A. Release of transfer ribonucleic acid from ribosomes - A G factor and guanosine triphosphate-dependent reaction. The Journal of biological chemistry. 1970;245(13):3346-51.

2. Valle M, Zavialov A, Sengupta J, Rawat U, Ehrenberg M, Frank J. Locking and unlocking of ribosomal motions. Cell. 2003;114: 123-134.
